# The challenge of avidity determination in SARS‐CoV‐2 serology

**DOI:** 10.1002/jmv.26863

**Published:** 2021-02-19

**Authors:** Georg Bauer, Friedhelm Struck, Patrick Schreiner, Eva Staschik, Erwin Soutschek, Manfred Motz

**Affiliations:** ^1^ Institute of Virology Medical Center, University of Freiburg Freiburg Germany; ^2^ Faculty of Medicine University of Freiburg Freiburg Germany; ^3^ Mikrogen GmbH Neuried Germany

**Keywords:** avidity, nucleoprotein, receptor‐binding domain, protective immunity, SARS‐CoV‐2, seasonal coronavirus

## Abstract

The serological responses towards severe acute respiratory syndrome coronavirus 2 (SARS‐CoV‐2) nucleoprotein, receptor‐binding domain (RBD), and spike protein S1 are characterized by incomplete avidity maturation. Analysis with varying concentrations of urea allows to determine distinct differences in avidity maturation, though the total process remains at an unusually low level. Despite incomplete avidity maturation, this approach allows to define early and late stages of infection. It therefore can compensate for the recently described irregular kinetic patterns of immunoglobulin M and immunoglobulin G (IgG) directed towards SARS‐CoV‐2 antigens. The serological responses towards seasonal coronaviruses neither have a negative nor positive impact on SARS‐CoV‐2 serology in general. Avidity determination in combination with measurement of antibody titers and complexity of the immune response allows to clearly differentiate between IgG responses towards seasonal coronaviruses and SARS‐CoV‐2. Cross‐reactions seem to occur with very low probability. They can be recognized by their pattern of response and through differential treatment with urea. As high avidity has been shown to be essential in several virus systems for the protective effect of neutralizing antibodies, it should be clarified whether high avidity of IgG directed towards RBD indicates protective immunity. If this is the case, monitoring of avidity should be part of the optimization of vaccination programs.

## Highlights

- Avidity maturation of immunoglobulin G (IgG) towards severe acute respiratory syndrome coronavirus 2 (SARS‐CoV‐2) antigens is distinct, but incomplete in most cases

- Nevertheless, avidity determination allows to differentiate between acute and past SARS CoV‐infection

- Avidity maturation is instrumental for differentiation between IgG responses towards SARS‐CoV‐2 and seasonal coronaviruses

- It is suggested to clarify whether high avidity is required for and indicative of protective immunity.

## 1 INTRODUCTION

The combination of direct detection of viral RNA or antigens with indirect recognition of infection through specific antibody determination is essential during the present pandemic. However, the analysis of data on severe acute respiratory syndrome coronavirus 2 (SARS‐CoV‐2) serology indicates that the humoral immune response towards SARS‐CoV‐2 does not always follow a regular pattern.^1^ Therefore, the classical differential determination of immunoglobulin M (IgM) and immunoglobulin G (IgG) responses was not found to be suitable to distinguish between acute and past SARS‐CoV‐2 infections. A model to explain variable IgM/IgG responses has been recently presented.^1^ In analogy to the resolution of other complicated serological constellations,^2^, ^3^, ^4^, ^5^, ^6^ avidity determination of specific IgG has been suggested as an alternative method for a clear differentiation between acute and past SARS‐CoV‐2 infections.^1^ This suggestion was based on our knowledge of avidity maturation as a regularly occurring, unidirectional process, and on the assumption that, like after other viral infections, avidity maturation would also take place during the humoral immune response towards SARS‐CoV‐2. Usually, the avidity maturation process starts from low avidity during acute infection and reaches high avidity in past infection.^1^ Avidity maturation is based on proliferation of IgG‐producing B cells, hypermutation of the variable part of immunoglobulin genes and clonal selection of B cells that express IgG of higher affinity on their surface than their neighboring cells.^7^, ^8^, ^9^, ^10^

Increasing affinity is reached through a constantly improved fit between the variable region of IgG and the respective epitope. This causes a faster reaction between IgG and its target epitope, as well as binding of IgG to this epitope with higher strength. As affinity, determined by these two reactions, is difficult to measure, the determination of the strength of the binding, termed “avidity,” is used as a meaningful marker. Avidity is representative for overall affinity, as the efficiency of the binding reaction, as well as its strength (avidity), depend on the same structural and mechanistic aspect, that is, the best fit between IgG and epitope. Avidity can be measured by the degree of release of IgG bound to its antigen by defined treatment with a chaotropic agent like urea. The comparision between an urea‐treated and untreated test allows to define the avidity index.

We used the *recomLine SARS‐CoV‐2* assay, a line assay developed for professional and commercial use. In this test highly purified recombinant SARS‐CoV‐2 nucleoprotein (NP), receptor‐binding domain (RBD), and S1 are arranged along with NP of four seasonal coronaviruses. This arrangement allows to quantify in one assay the IgG responses and the avidity of the determined IgG towards all the implemented antigens.

The use of this test system, led to the surprising result that avidity maturation of IgG towards SARS‐CoV‐2 antigens was frequently incomplete, and that incomplete avidity maturation seemed to be due to a discontinuous kinetics of avidity maturation rather than to a too short time of observation.^11^ Therefore, even several months after infection, most of the sera from coronavirus disease 2019 (COVID‐19) patients showed immature avidity of IgG towards SARS‐CoV‐2 antigens. Interestingly, the degree of avidity maturation was higher in patients with more severe disease. This finding is in line with several reports on a relative increase in avidity towards SARS‐CoV‐2 antigens in hospitalized patients ^12^, ^13^, ^14^ and corresponds to increased IgG titers towards SARS‐CoV‐2 in patients with more severe disease.^15^, ^16^

This remarkable pattern of incomplete avidity maturation of SARS‐CoV‐2 specific IgG poses several diagnostic problems that are resolved this manuscript.

## 2 MATERIALS AND METHODS

### 2.1 Patients and sera

#### 2.1.1 SARS‐CoV‐2‐positive sera

Sera from adult outpatients (18–65 years) with clinical signs of COVID‐19 and SARS‐CoV‐2 infection confirmed by polymerase chain reaction were collected after a call in the Munich area for voluntary donation of a serum sample. The samples were drawn by family doctors after explicit written consent of the volunteers. The logistic support of Mikrogen GmbH collected the sera and relevant information on the patients.

The samples were then anonymized and tested by the Research and Development group of Mikrogen GmbH, using the newly established *recomLine*SARS‐CoV‐2 line assay. For the testing personel and for the first author (G.B.) who analyzed the data, no personal data were available, except on gender, clinical symptoms of the patients, the data of extraction of the sera and the time between onset of clinical symptoms and extraction of the sera. These data are listed in Table S1.

#### 2.1.2 SARS‐CoV‐2‐negative sera

Three hundred anonymized plasma samples from healthy adult blood donors were purchased from the Bavarian Red Cross. The blood donor sera were collected before the outbreak of the SARS‐CoV‐2 pandemic, that is, before November 2019. They have been assayed to determine the specificity of the recomLine SARS‐CoV‐2.

Sera were stored at −20°C until they were tested in the immunoassays.

#### 2.1.3 Immunoblot assay

A. Production of *recomLine* SARS‐CoV‐2 nitrocellulose strips: Individual concentrations of purified recombinant antigens NP, RBD, S1 of SARS‐CoV‐2, as well as NP of 229E, NL63, OC43, HKU1 were applied directly onto nitrocellulose membranes in separate lanes. Production was standardized and the resultant strips were evaluated (see Supplementary Materials for details), resulting in the CE‐marked product #7374 of Mikrogen GmbH.

B. Procedure of the line immunoassay: The reactivity of 1:100 dilutions of serum antibodies against the recombinant antigens was detected with peroxidase‐labeled anti‐human IgG antibody and the use of precipitating tetramethylbenzidine. The first incubation of serum and test strips was for 1 h, followed by three washing steps with buffer. The incubation of the strips with peroxidase‐labeled anti‐human IgG antibody was for 45 min, followed by three washing steps. Treatment with tetramethylbenzidine was for 8 min.

The line immunoassays were carried out in a semiautomatic processor Dynablot (Dynex Technologies GmbH) with manual serum pipetting according to instruction manual provided by Mikrogen GmbH. An Epson J371A scanner (Epson) and recomScan software (Mikrogen GmbH) were used according to the instruction manuals.

C. Avidity determination: sera were incubated for 1 h with the recomLine SARS‐CoV‐2 test strips in duplicate; then both replicates incubated for 5 min with wash buffer, and one assay was incubated in wash solution, while the parallel assay replicate was treated with the indicated concentrations of urea for 3 min; after three additional washing steps both assay replicates were processed with anti‐human IgG antibody labeled with peroxidase and detected as outlined above to describe the line immunoassay procedure. The gray intensity area output by recomScan on the urea treated test strip was divided by the gray intensity of the parallel assay replicate to determine the avidity index arithmetically.

### 2.2 Statistics

Due to the established professional performance of the recomLine SARS‐CoV‐2 line assay, all determinations were performed under conditions of routine diagnostics, that is, sera were tested individually in single assays. Three sera were tested in repeat experiments, using variable concentrations of urea both in the initial and the repeat experiment. No statistical significant difference was observed between the initial and the repeat experiment.

The data analysis by G. Bauer was performed on the basis of raw data.

The Yates continuity corrected *χ* ^2^ test (two‐sided) was used for the statistical determination of significances (*p* < 0.01 = significant; *p* < 0.001 = highly significant).

## 3 RESULTS

### 3.1 Kinetics of avidity maturation of IgG towards NP, receptor‐binding domain, and S1

**Figure 1:**
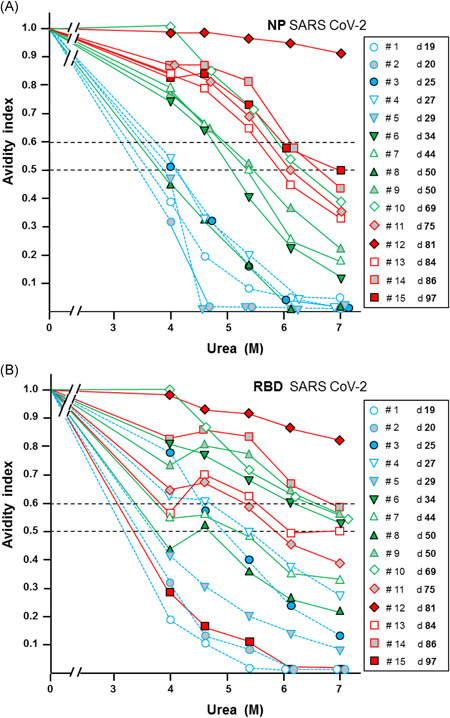
Avidity determination of immunoglobulin G (IgG) towards nucleoprotein (NP) and receptor‐binding domain of the S protein (RBD) in 15 sera from patients with coronavirus diesease 2019 (COVID‐19). Avidity determination was performed with the indicated concentrations of urea for IgG directed towards severe acute respiratory syndrome coronavirus 2 (SARS‐CoV‐2) (A) NP or (B) RBD in 15 sera of patients with COVID‐19 and SARS‐CoV‐2 infection proven by polymerase chain reaction (PCR). The sera had been taken at varying times after the onset of disease, as indicated in the figure. Dashed lines indicate the level between low avidity (avidity index < 0.5), borderline avidity (avidity index between 0.5 and 0.6) and high avidity (avidity index >0.6). With the exception of one serum, the majority of sera exhibited IgG of low or borderline avidity for IgG towards (A) NP and (B) RBD, confirming the immature avidity response after SARS‐CoV‐2 infections. Though the avidity indices obtained at 7 M urea are mostly in the borderline and low avidity range, the titration with varying concentrations of urea visualizes the individual differences in a more pronounced and characteristic mode. This refined measurement therefore allows to follow subtle changes of avidity in defined cases of analysis. The grouping of the sera with respect to time after onset of disease showed that the increase in avidity of IgG directed towards NP seemed to occur in a rather coordinate mode, whereas the increase in avidity of IgG towards RBD was characterized by a larger degree of variability

Sera from COVID‐19 patients taken 19–97 days after onset of disease were tested for avidity of IgG directed towards SARS‐CoV‐2 nucleoprotein (NP), RBD of spike protein and spike protein S1. Instead of standard testing with 7 M urea, increasing concentrations of urea (4–7 M) were used. This approach allows for a more refined determination of the binding strength of specific antibodies than previous measurements with the standard concentration of 7 M urea. The resulting titration curves showed a strong variation in the degree of avidity. Importantly, only 1 serum out of 15 had indeed high avidity IgG towards NP, though the time span between onset of disease and acquisition of the sera ranged up to more than 3 months past onset of disease (Figure 1A). Though 14 sera did not show high avidity of IgG directed towards SARS‐CoV‐2 NP (as defined by an avidity index higher than 0.5 at 7 M urea), the grouping of the sera according to the time of their recovery allowed to recognize a nearly uniform increase of avidity between these groups. The test for avidity of IgG directed towards RBD (Figure 1B) and S1 (Figure S1) showed that the serum with high avidity towards NP also exhibited high avidity towards RBD and S1. Otherwise, all sera were either in the low avidity range or the border zone of intermediate avidity. The grouping of avidity determinations for IgG towards RBD and S1 according to the time of extraction showed a high degree of variability. These findings confirm the recently determined incomplete avidity maturation during the serological response towards SARS‐CoV‐2 antigens and extend the significance of this finding due to the more refined measurement.

**Figure 2:**
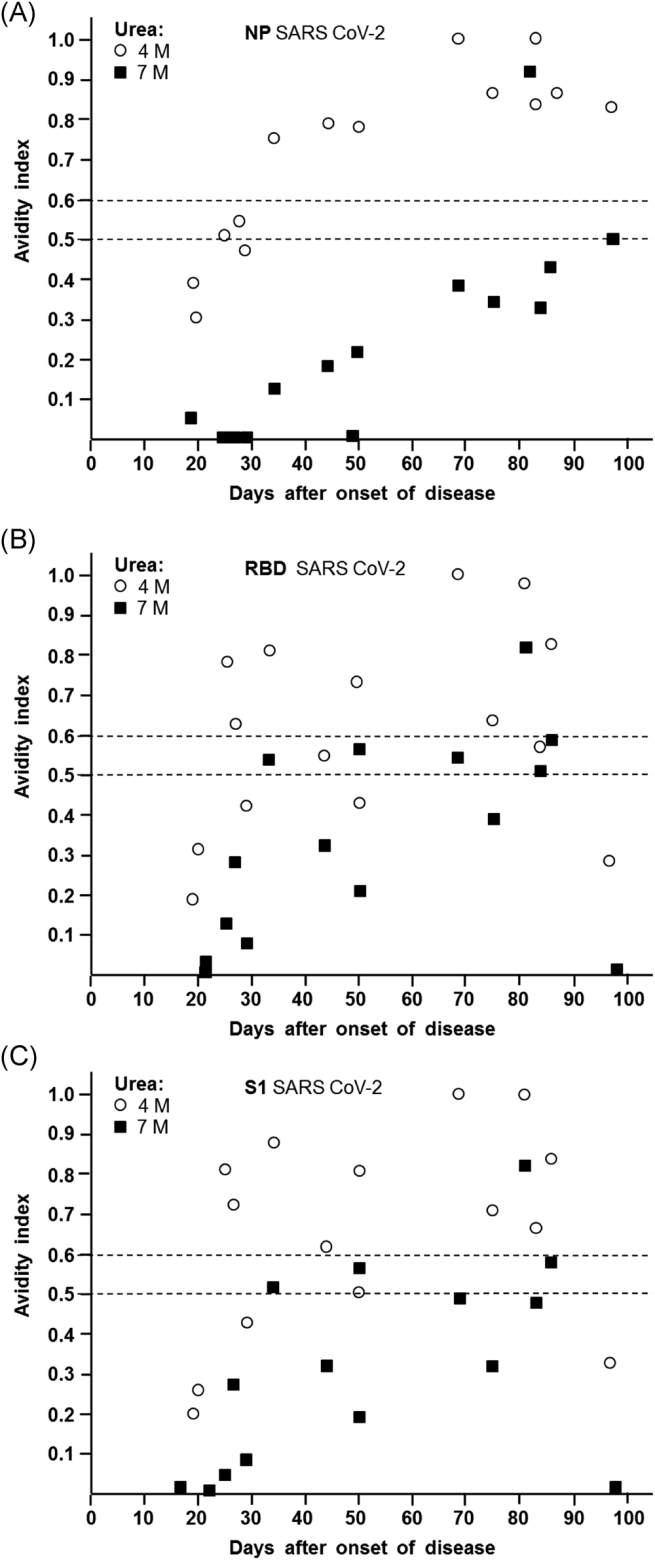
Dependency of avidity indices of IgG towards NP, RBD, and S1 of SARS‐CoV‐2 on the time after onset of disease. Avidity indices were taken from the experiment described in Figure 1 and Supplementary Figure 1, and were plotted against the time between onset of disease and serum acquisition. The avidity indices obtained after treatment with 4 and 7 M urea are shown for IgG directed towards (A) NP, (B) RBD, and (C) S1. (A) The curves obtained for 4 and 7 M urea are clearly separated (*p* <0.001). Though 14/15 sera remain in the low avidity range for IgG towards NP when 7 M urea had been applied, an avidity index of 0.3 seems to be appropriate to distinguish between sera taken before or after 50 days after onset of disease (*p* = 0.002). For treatment with 4 M urea, an avidity index of 0.5 seems to be suitable for discrimination between sera taken before or after 30 days after onset of disease, though this discrimination is statistically weak (*p* = 0.05). (B, C) Due to the higher variability of the avidity indices for IgG directed towards RBD and S1, compared to the values obtained for NP under A, the overlap between the curves for 4 and 7 M urea is strong. In addition, the high variability of the avidity indices and the relative low number of cases does not allow to define a significant point of differentiation between acute and past infection. IgG, immunoglobulin G; NP, nucleoprotein; RBD, receptor‐binding domain; SARS‐CoV‐2, severe acute respiratory syndrome coronavirus 2

When the avidity indices, which had been determined with different concentrations of urea and for IgGs directed towards the three different antigens in the experiments described in Figure 1 and Figure S1, were plotted against the time of onset of disease, consistent patterns were seen (Figure 2). Though avidity maturation remains largely incomplete, as seen by the low avidity indices obtained with 7 M urea, a certain degree of avidity maturation can nevertheless been seen with time. In the case of IgG towards NP, the continuous increase could be taken as a measure of the time point relative to the time of onset of disease (Figure 2A). When the avidity index of IgG towards NP was determined with 4 M urea, a distinction of lower avidity before Day 30 and higher avidity thereafter was also possible. Though the increase of avidity indices for IgG towards RBD and S1 are also showing a strong tendency for partial maturation with time (Figure 2B,C), their much higher degree of variability compared to IgG towards NP would not allow a useful determination of the time point related to the onset of disease or infection. Due to the high degree of variability, there was a strong overlap between the curves obtained by treatment with 4 versus 7 M urea.

**Figure 3:**
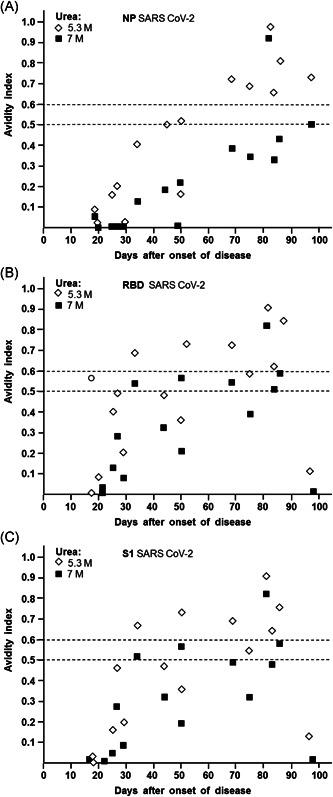
Dependency of avidity indices of IgG towards NP, RBD, and S1 of SARS‐CoV‐2 on the time after onset of disease. Avidity indices were taken from the experiment described in Figure 1 and Figure S1, and were plotted against the time between onset of disease and serum acquisition. The avidity indices obtained after treatment with 5.3 and 7 M urea are shown for IgG directed towards (A) NP, (B) RBD, and (C) S1. (A) The curves obtained for 4 and 7 M urea are clearly separated (*p* < 0.001). For treatment with 5.3 M urea, an avidity index of 0.5 seems to be suitable for discrimination between sera taken before or after 50 days after onset of disease (*p* =0.015). However, as already outlined in Figure 2A, the use of 7 M urea and an avidity index cut‐off of 0.3 is more significant and therefore better suitable for practical use. (B, C) Due to the higher variability of the avidity indices for IgG directed towards RBD and S1, compared to the values obtained for NP under A, the overlap between the curves for 5.3 and 7 M urea are strong. In addition, the high variability of the avidity indices and the relative low number of cases does not allow to define a significant point of differentiation between acute and past infection. IgG, immunoglobulin G; NP, nucleoprotein; RBD, receptor‐binding domain; SARS‐CoV‐2, severe acute respiratory syndrome coronavirus 2

An analogous picture was seen when the avidity treatment with 7 M urea were compared to 5.3 M urea (Figure 3). Again, the curves for IgG towards NP were separated from each other and their continuous increase allowed for calibration of individual sera tested (Figure 3A), whereas the curves for IgG towards RBD or S1 seemed to be less suitable (Figure 3B,C).

The relative increase in avidity with time was not paralleled by an increase in IgG concentrations, which were scattered along the time axis (Figure S2). Therefore, there was also no good correlation between IgG concentration and avidity index

The difference between the avidity indices of IgGs towards NP and RBD and their variability are illustrated in Figure S3.

This study has been performed with a relatively low number of sera (*n* = 15), but applied more extensive analysis of avidity than could be performed in routine diagnostics. To verify or falsify our conclusions on the specific features of avidity of IgG directed towards SARS‐CoV‐2 NP, RBD and S1, a larger number of sera (*n* = 93) from SARS‐CoV‐2‐infected COVID‐19 outpatients (*n* = 70) were tested for avidity under conditions of routine avidity testing, using 7 M urea. Figures S4 and S5 show the verification of our conclusions through this follow‐up experiment. It was confirmed that SARS‐CoV‐2 infection is characterized by frequent incomplete avidity maturation and therefore high avidity as determined by application of 7 M urea seems to be the exception. Despite this unique feature, the increase in avidity of IgG towards NP nevertheless allows for a discrimination between acute and past infections.

### 3.2 The potential impact of IgG towards seasonal coronaviruses on SARS‐CoV‐2 serology

As infections with seasonal coronaviruses are occurring repeatedly at certain intervals,^17^, ^18^ a potential positive interference of IgG towards antigens of seasonal coronaviruses on SARS‐CoV‐2 serology is a major concern. To address this aspect, the line assays for the determination of the serological response towards SARS‐CoV‐2 NP, RBD and S1 have been complemented with NP of the four major seasonal human coronaviruses, that is, 229 E, NL63, OC43, and HKU1. This approach allows a direct determination of IgG towards NP of seasonal coronaviruses and SARS‐CoV‐2 and the respective avidities, in the same assay system.

**Figure 4:**
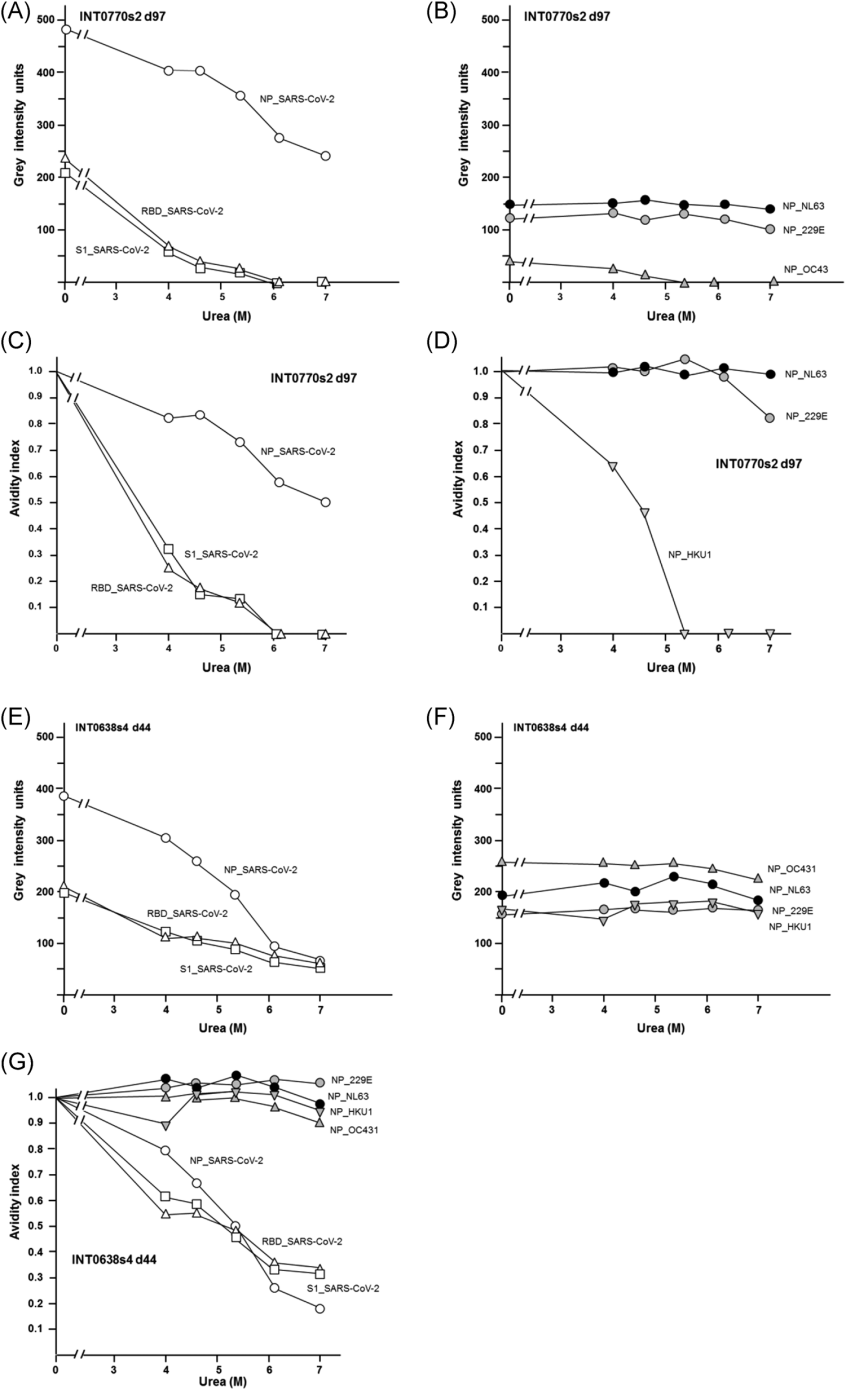
Comparison of gray intensity units and avidity indices obtained for IgG directed towards NP, RBD, and S1 of SARS‐CoV‐2 and NP of four seasonal coronaviruses. Sera taken from two COVID‐19 patients at (A–D) Day 97 or (E–G) Day 44 after onset of disease were tested in the recomLine SARS‐CoV‐2 assay, treated without urea or with increasing concentrations of urea, as indicated in the Figure. Gray intensity units (A, B, E, and F) and calculated avidity indices (C,D, and G) are presented. (A) The patient with the ID INT0770 shows a high gray intensity units for IgG towards NP of SARS‐CoV‐2 and a moderate reduction of gray intensity units after urea treatment, whereas gray intensity units for IgG towards RBD and S1 are lower, close together, and strongly reduced by urea treatment. (B) The patient also shows IgG towards NP of the seasonal coronaviruses NL63, 229 E, and OC43 in a much lower range of gray intensity units as measured for IgG towards NP of SARS‐CoV‐2 under A. The IgG towards NP of NL63 and 229 E is not significantly affected by urea treatment, whereas the very low concentration of IgG towards NP of OC43 is completely removed by urea. The analysis of the avidity indices (C,D) shows borderline avidity for IgG towards NP of SARS‐CoV‐2 and very low avidity for IgG towards RBD and S1 of SARS‐CoV‐2, whereas the avidity indices for IgG towards NP of NL63 and 229 E are very high, and very low for IgG towards NP of OC43. These data show that the IgG response towards NP of SARS‐CoV‐2 cannot be explained by cross‐reaction caused by IgG towards seasonal coronaviruses, as the concentration of IgG towards NP is lower than that of IgG towards NP of SARS‐CoV‐2 and the avidity indices of IgG towards NP of SARS‐CoV‐2 and those of IgG towards NP of the seasonal coronaviruses are not matching. The data shown for a second patient (ID INT0638) under (E–G) confirm these conclusions, as the gray intensity values measured for NP towards (E) SARS‐CoV‐2 are much higher than those for four (F) seasonal coronaviruses, and IgG towards NP of SARS‐CoV‐2 shows low avidity, whereas (G) the IgG towards the NPs of all four seasonal coronaviruses is of very high avidity. COVID‐19, coronavirus disease 2019; IgG, immunoglobulin G; NP, nucleoprotein; RBD, receptor‐binding domain; SARS‐CoV‐2, severe acute respiratory syndrome coronavirus 2

As shown in Figure 4A, the relative gray intensity values of a serum taken from a COVID‐19 patient as late as 97 days after onset of disease showed a value of nearly 500 units, which decreased substantially with the concentration of urea used in the assay. The gray intensity values for IgG towards RBD and S1 were substantially lower and also decreased with urea treatment. The calculation of the avidity indices (Figure 4C) showed an intermediary avidity for IgG towards NP and low avidity for IgG towards RBD and S1. The gray intensity values obtained for IgG towards NP of three seasonal coronaviruses (Figure 4B) were markedly lower than the corresponding value of IgG towards SARS‐CoV‐2 NP. The higher gray intensity values of IgG towards SARS‐CoV‐2 NP is the first clear argument against cross reaction between IgG towards NP of seasonal coronaviruses with NP of SARS‐CoV‐2 being the reason for the signal measured. The determination of the avidity indices of IgG towards NP of the seasonal coronaviruses (Figure 4D) was the second counter argument, as two of the IgGs showed very high avidity, whereas one showed very low avidity. In contrast, the avidity of IgG towards SARS‐CoV‐2 NP was of intermediary avidity. This example shows that the parallel determination of IgG towards SARS‐CoV‐2 antigens and those of seasonal coronaviruses opens the chance to clearly differentiate these obviously none‐overlapping responses.

Figure 4E–G demonstrates a second example. Again, the gray intensity value of IgG towards NP of SARS‐CoV‐2 was high and of low avidity, whereas the IgG response towards NP of four seasonal coronaviruses was uniformly lower, but of high avidity.

**Figure 5:**
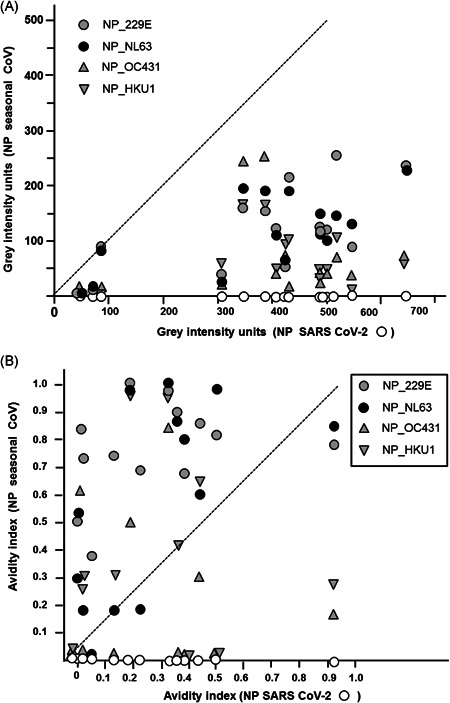
Lack of correlation between the gray intensity units and avidity indices of IgG towards NP of SARS‐CoV‐2 NP of four seasonal coronaviruses. (A) The gray intensity units for IgG towards NP of SARS‐CoV‐2 (as determined in Figure 1) were plotted against the gray intensity units of IgG towards NP of the seasonal coronaviruses 229 E, NL63, OC43, and HKU1 which had been determined in the same assays. With one exception, the gray intensity units obtained for IgG towards NP of seasonal coronaviruses was always much lower than the values obtained for IgG towards NP of SARS‐CoV‐2 (*p* < 0.001), thus excluding that the values obtained for SARS‐CoV‐2 were due to cross‐reaction with IgG directed towards NP of the seasonal coronaviruses. (B) The avidity indices corresponding to the data presented under A, determined by treatment with 7 M urea versus untreated controls, were plotted (NP SARS‐CoV‐2 vs. NP of seasonal coronaviruses). The data show that only in five cases the avidity index of one of the seasonal coronaviruses was matching the avidity index of IgG towards NP of SARS‐CoV‐2, whereas the avidity indices of IgG towards the NPs of seasonal coronaviruses were lower in 6 cases and higher in 30 cases, compared to the avidity of IgG towards NP of SARS‐CoV‐2. The data from Figure 5A,B indicate that in the vast majority of cases the IgG response towards NP of SARS‐CoV‐2 cannot be explained by crossreactive IgG directed towards NPs of the four seasonal coronaviruses tested. IgG, immunoglobulin G; NP, nucleoprotein; SARS‐CoV‐2, severe acute respiratory syndrome coronavirus 2

Based on formal logics, the IgG directed towards NP of seasonal coronaviruses is therefore excluded as cause for the IgG response towards SARS‐CoV‐2 NP: More examples, confirming that IgG towards NP of seasonal coronaviruses cannot explain the values obtained for IgG towards SARS‐CoV‐2 NP are shown in Supplementary Figure 6. This conclusion is further substantiated through the comparison of the gray intensity values and avidity indices towards SARS‐CoV‐2 NP in 15 sera and the corresponding values of IgG towards seasonal coronaviruses. As shown in Figure 5, there was no correlation between IgGs towards SARS‐CoV‐2 NP and NP of the four seasonal coronaviruses, both with respect to antibody concentration (A) and avidity (B). With one exception, the gray intensity values of IgGs directed towards the NPs of the seasonal coronaviruses were markedly lower than those of IgG towards SARS‐CoV‐2 NP, whereas most sera showed higher avidity indices for the NPs of seasonal coronaviruses than for NP of SARS‐CoV‐2. This indicates that the serology of seasonal coronaviruses does not overlap with with SARS‐CoV‐2 serology and therefore cannot interfere with it.

The preceding figures have shown that the serological response towards SARS‐CoV‐2, as well as towards seasonal coronaviruses frequently may enface incomplete avidity maturation, in line with our previous findings.^11^ The avidity determination in individual sera therefore does not always allow to immediately distinguish between (i) immature avidity and (ii) low avidity due to just ongoing acute infection. Careful quantitation of avidity in combination with testing a subsequent serum can, however, easily resolve such questions. In several cases, it was shown that, though IgG towards SARS‐CoV‐2 remained in the lower avidity range, a discrete increase of avidity with time was apparent. Low avidity IgG directed towards seasonal coronaviruses detectable in the same serum remained at the same level of avidity with time, indicating that it represented a condition of incomplete avidity maturation in a previous infection (data not shown).

### 3.3 Specificity of SARS‐CoV‐2 serology performed with the line assay

**Figure 6:**
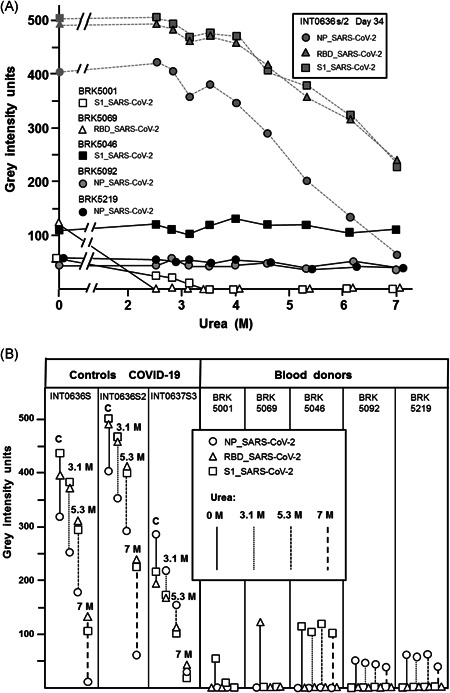
Evaluation of the specificity of the SARS‐CoV‐2 IgG determination. (A) 5 sera out of 300 sera from healthy blood donors that had been collected three months before the pandemic gave a low positive signal (gray intensity units) towards individual SARS‐CoV‐2 antigens in the recomLine SARS‐CoV‐2 IgG test. In contrast, a reference serum from a PCR‐confirmed COVID‐19 patients showed high gray intensity units towards all three SARS‐CoV‐2 antigens tested (NP, RBD, S1). Treatment with increasing concentrations of urea up to 3.5 M did not affect the binding of IgG from the truely SARS‐CoV‐2‐positive serum, but completed removed IgG of two of the blood donor sera from the antigen (RBD or S1), whereas three blood donor sera remained unaffected under these conditions. Further increase in the urea concentration showed low avidity of IgG towards SARS‐CoV‐2 NP, borderline avidity of IgG towards SARS‐CoV‐2 RBD and S1, and very high avidity of three of the blood donor sera towards either S1 (one serum) or NP (two sera). (B) Part B summarizes the findings for three truly positive reference sera and the reactive sera from uninfected blood donors. The figure shows that the positively reacting sera from blood donors only showed reactivity towards one of the SARS‐CoV‐2 antigens, whereas the specific control sera reacted with all three antigens (in line with findings for all SARS‐CoV‐2 positive sera tested by us so far). The reactivity of two of the blood donor sera was removed with very low concentrations of urea (2.5 M), pointing to a weak cross‐reaction. The other three sera showed high avidity IgG towards one antigen, despite their low concentration, pointing to a cross‐reactive epitope shared between seasonal coronaviruses and SARS‐CoV‐2. COVID‐19, coronavirus disease 2019; IgG, immunoglobulin G; NP, nucleoprotein; PCR, polymerase chain reaction; SARS‐CoV‐2, severe acute respiratory syndrome coronavirus 2

The specificity of a serological test is determined by the percentage of correctly diagnosed negative cases in a population of negatively defined test samples. To determine the specificity of the SARS‐CoV‐2 IgG test, 300 serum samples from healthy adult blood donors, obtained several months before the pandemic, were tested. Five serum samples from 300 gave a positive result repeatedly. In all cases, the positive result was in the very low range of gray intensity units (Figure 6A). In two cases the values were around 100, in three cases the values were very close to the cut‐off value of about 50 units. Besides their low reactivity, all five positive sera had in common that they only showed reactivity towards one of the SARS‐CoV‐2 antigens Two sera were directed towards NP, two towards S1 and one towards RBD. In two of the sera, extremely low concentrations of urea (2.5–3 M) were sufficient to remove the bound IgG from the assay, whereas IgG of specific anti‐SARS‐CoV‐2 serum even with low avidity was not significantly affected at such low urea concentrations. This finding points to a relative week cross reactivity between an IgG directed towards a saisonal coronavirus and a related, but distinct epitope on an analogous SARS‐CoV‐2 antigen. The remaining three sera showed very high avidity towards the SARS‐CoV‐2 antigen even at 7 M urea. This finding is best explained by the reactivity of IgG of high avidity directed towards an epitope on the antigens of a seasonal coronavirus with a perfectly matching epitope on a SARS‐CoV‐2 specific antigen. These data (summarized in Figure 6B) illustrate that quantitative avidity determination with variable urea concentrations can help to recognize positive signals as false positives relatively easily. It thus finally increases the specificity of the serological test used and thus should ensure correct serodiagnosis also in more complicated serological cases.

## 4 DISCUSSION

### 4.1 Low avidity of IgG towards SARS‐CoV‐2 antigens

The repeated findings on low avidity of IgG directed towards SARS‐CoV‐2 antigens, even several months after onset of clinical symptoms^11^, ^13^, ^14^, ^19^, ^20^, ^21^; are unique in viral serodiagnostics so far. Though Luo et al.^12^ stated that there was a strong correlation between avidity or IgG towards RBD and days after onset of symptoms, their findings are also supporting the findings on low avidity of IgG towards SARS‐CoV‐2 antigens, as the authors had used the very low concentration of 3 M urea in their study. Navarro et al.^22^ stated an increase in avidity with time. This study is difficult to interprete in the context of the quantitative studies on avidity, as IgM and IgG avidity were measured without differentiation and their assay was only performed as qualtitative estimation.

Our data, in line with our present study, clarify that low avidity of IgG towards SARS‐CoV‐2 NP, RBD, and S1 were not due to theoretically conceivable test‐inherent problems like increased sensitivity of the test antigen towards the denaturing potential of urea, as high avidity was repeatedly determined in a small percentage of sera. These sera with high avidity IgG represent the essential positive control of the test system. According to our data, low avidity of IgG towards SARS‐CoV‐2 antigens was due to incomplete avidity maturation.^11^ Kinetic analysis showed that the breakpoint of decreasing and even declining IgG responses correlated with the point of interrupted avidity maturation.^11^ As a consequence of discontinuous avidity maturation, low or intermediate avidity indices remain stable at their level over time. Without the knowledge about discontinuous avidity maturation of IgG towards SARS‐CoV‐2, such findings might be misinterpreted as being indicative of acute infection with SARS‐CoV‐2. A similar scenario has been found for the immune response towards seasonal coronaviruses.^11^

Declining IgG responses towards SARS‐CoV‐2, including those for neutralizing IgG directed towards the receptor binding domain RBD, have been determined by several other groups.^15^, ^20^, ^23^, ^24^ They point to a central problem after natural SARS‐CoV‐2 infection: Waning antibody levels most likely indicate the lack of protective immunity and thus might prevent the establishment of effective herd immunity.

The method to titrate the urea concentration allowed a rather precise characterization of the avidities of the sera study and confirmed our previous findings that had been based on the effects of the “discriminative” urea concentration of 7 M. The use of variable urea concentrations was suitable to determine even marginal kinetic increases in avidity maturation, as well as cessation of the avidity maturation process.

One of the practical goals of this study was to establish avidity determination of SARS‐CoV‐2 specific IgG as a method that would allow an unequivocal differentiation between acute and past SARS‐CoV‐2 infection. On the first glance, the incomplete avidity maturation seemed to indicate that this goal cannot be easily achieved. However, the kinetic analysis in Figures 3 and 4 demonstrates, that a discrimination between acute and past infection can nevertheless be reached, based on IgG towards NP and the use of either 4, 5.3, or 7 M urea. Depending on the urea concentration used, “cut‐off values” of avidity for the distinction between early and late phases of infection or disease have to be defined independently. For example, a cut‐off of 0.3 easily helps to discriminate between a begin of disease within or after 50 days (Figure 2A), when 7 M urea was used in the test system and IgG towards NP was analyzed. Due to their higher variability, the IgG responses towards RBD and S1 and their avidity are less suitable for discrimination between acute and past infection. However, the role of these IgGs is confirmatory for true positive anti‐SARS‐CoV‐2 responses and of potential importance for the determination of protective immunity, as discussed below.

### 4.2 SARS‐CoV‐2 serology is not largely affected by seasonal coronaviruses

The serological responses towards the seasonal coronaviruses 229 E, NL63, OC43 and HKU1 were neither positively nor negatively interfering with the serological results obtained for SARS‐CoV‐2. With one exception, the concentrations of IgG directed NP of seasonal coronaviruses were always much lower than the concentrations of IgG measured for SARS‐CoV‐2 in the same sera, excluding the theoretical assumption that the values obtained for SARS‐CoV‐2 were due to cross‐reactive antibodies induced by seasonal coronaviruses. This argument is further strengthened by the finding that the avidity indices of IgGs towards NPs of seasonal coronaviruses do not match the avidity indices found for IgG towards NP of SARS‐CoV‐2. In addition, the sera obtained from healthy blood donors that had donated serum before the SARS‐CoV‐2 pandemic, showed that false positive results for SARS‐CoV‐2 IgG were extremely rare. They could be easily differentiated from true positives, as (i) the antibody concentration in these cases was extremely low, (ii) the false positive responses were directed towards isolated antigens of SARS‐CoV‐2, whereas true positive results were uniformly directed towards all three antigens tested,^11^ (iii) two of the false positives were converted to negative by extremely low concentrations of urea, pointing to a crossreaction of questionable significance and (iv) three false positives were recognized by the previous parameters of low concentration and reaction towards one antigen only, and in addition showed very high avidity, despite their low titer. The latter findings were indicative for a cross‐reactive epitope on seasonal coronaviruses and SARS‐CoV‐2.

Interestingly, our data also confirm that not only SARS‐CoV‐2, but also seasonal coronaviruses, seem to elicit a humoral immune response that is frequently characterized by incomplete avidity maturation. A closer look at the avidity data published for SARS CoV‐1 ^25^ reveals that the maturation curve for IgG towards this first SARS CoV has been established with 4 M urea, rather than with the sharply discriminative concentration of 7 M. Therefore it can be concluded that SARS CoV‐1 also seems to induce an immune response that is characterized by low avidity. These findings allow the speculation that restriction to low avidity antibodies might be part of the biological strategy of coronaviruses in general—ensuring repeated waves of reinfection.^17^, ^18^

### 4.3 Avidity and protection towards infection

A growing body of evidence shows that avidity maturation plays a central and dominant role for antibody‐mediated protection towards viral infections. Protection towards viral infections fails if avidity maturation of IgG directed towards the respective viruses is failing.^26^, ^27^, ^28^, ^29^, ^30^, ^31^, ^32^, ^33^, ^34^, ^35^ Supporting this view, vaccination studies for Simian human immunodeficiency virus have shown a strong correlation between the avidity of the IgG towards the envelope protein and protection towards viral infection.^36^, ^37^

These data show convincingly and for a broad variety for viruses, that binding of antibodies to a specific target was only protective if the antibodies had reached high avidity. Therefore, we propose that the goal of vaccination programs towards SARS‐CoV‐2, should be to reach an IgG response that (i) specifically targets relevant surface structures of SARS‐CoV‐2, such as RBD, (ii) is sufficiently high in its titer and, (iii) has acquired high avidity. High avidity of such truly neutralizing IgG should also ensure the generation of corresponding memory cells with their potential to elicit an efficient protective effect even at later time points.^38^ The first attempts to generate an efficient vaccine seem to be very promising.^39^ It is exciting to see that vaccination towards SARS‐CoV‐2 can induce IgG responses that are much higher than those generated by natural infection.^40^, ^41^ Based on the mechanism of avidity maturation, with its many cycles of mutation and clonal selection, the prolonged availability of antigen seems to be an absolute requirement for proper avidity maturation.^32^, ^42^, ^43^, ^44^ The mode of vaccination should fulfill this requirement. Further analysis is required to clarify the potential role of high avidity for protective immunity.^45^ The test system presented in this manuscript seems to represent a promising tool for the resolution of this important issue. Provided our conclusions can be verified, avidity determination has a good chance to be instrumental for optimization of the mode of vaccination and to allow the determination of protective immunity in individual cases that require certainity of their state of protection. Please find more details under Supplementary Discussion.

## CONFLICT OF INTERESTS

E. Soutschek and M. Motz are owners of Mikrogen GmbH. E. Soutschek is the present CEO of Mikrogen GmbH. F. Struck, E. Stachik and P. Schreiner are employees of Mikrogen GmbH. The determination of avidity of antibodies through immunoblots has been patented by Mikrogen GmbH (WO 00/54055; PCT/EP00/01883). In addition, a patent application for a method to determine the avidity of antibodies towards SARS‐CoV‐2 is pending (EP 2019/2550). G. Bauer is a member of the Medical Faculty of the University of Freiburg. He is the inventor of WO 00/54055; PCT/EP00/01883 and one of the coinventors of EP 2019/2550.

## AUTHORS CONTRIBUTIONS

Friedhelm Struck, Patrick Schreiner, Eva Staschik, Erwin Soutschek, and Manfred Motz: Development and evaluation of the test system, organizing and supervising testing in‐house, documentation and discussion of data, and commenting the manuscript. Georg Bauer: Analysis of raw data, generation of the graphs, conceptualization, and writing the manuscript.

## Supporting information

Supporting information.

## Data Availability

All data used for this manuscript are documented in the manuscript and its supplement.
