# Supplementary material for: The challenge of avidity determination in SARS‐CoV‐2 serology
Source: J Med Virol. 2021 Feb 19;93(5):3092–104. doi: 10.1002/jmv.26863 (PMC8013859; doi:10.1002/jmv.26863)
Supplement: Supplementary file 1 — Supporting information. [file JMV-93-3092-s001.docx]

**Supplementary Material**

The challenge of avidity determination in SARS-CoV-2 serology

Georg Bauer ^1, 2,^ **^*^**, Friedhelm Struck ^3^, Patrick Schreiner ^3^, Eva Staschik ^3^,

Erwin Soutschek ^3^, Manfred Motz ^3^

1) Institute of Virology, Medical Center - University of Freiburg

2) Faculty of Medicine, University of Freiburg, Freiburg, Germany

3) Mikrogen GmbH, Neuried, Germany

**^*^** Corresponding author (Email: georg.bauer@uniklinik-freiburg.de)

Supplementary Material contains

1. Patients and sera pages 2-3
2. Supplementary Methods pages 3-4
3. Supplementary Results pages 4-14
4. Supplementary Discussion pages 14-21
5. Supplementary References pages 22-25

**A. Patients and sera**

15 sera from 11 adult outpatients with clinical signs of COVID-19 and SARS-CoV-2 infection confirmed by positive PCR results were collected after a call in the Munich area for voluntary donation of a serum sample. The samples were drawn by family doctors after explicit written consent of the volunteers.

The available data are summarized in Supplementary Table 1.

Supplementary Table 1: Patients and sera used in this study

| Identification  Number. | Working  number | Days after onset of disease | PCR | Gender | Reported  Symptoms |
| --- | --- | --- | --- | --- | --- |
| INT0637 | # 1 | 19 | + | f | ST, F, H, LS |
| INT0639 | # 2 | 20 | + | f | ST, F, H, LS |
| INT0587 | # 3 | 25 | + | m | mild symptoms |
| INT0636 a | # 4 | 27 | + | m | ST, F, H, LS |
| INT0636 b | # 6 | 34 |  |  |  |
| INT0636 c | # 9 | 50 |  |  |  |
| INT0602 a | # 5 | 29 | + | ud | ST, F, LS |
| INT0602 b | # 8 | 50 |  |  |  |
| INT0638 a | # 7 | 44 | + | m | ST, F, H |
| INT0638 b | # 13 | 84 |  |  |  |
| INT0759 | # 10 | 69 | + | f | ST |
| INT0682 | # 11 | 75 | + | m | mild symptoms |
| INT0760 | # 12 | 81 | + | m | F, pneumonia |
| INT0771 | # 14 | 86 | + | m | ST, F |
| INT0770 | # 15 | 97 | + | f | mild symptoms |

Table 1 summarizes the patients and sera used in the main part of this study. The identification numbers were given to the patients. If more than one serum was drawn, the addition a, b, c has been made. The sera were sorted according to the time after onset of disease and received a working number. All patients were outpatients, had been tested positive for SARS-CoV-2 by PCR and had symptoms that did not require hospitalization. Specific symptoms: ST = sore throat; F = fever; H = headache; LS = loss of smell. ud = undefined.

For the determination of specificity of our test system, 300 anonymized plasma samples from healthy adult blood donors were purchased from the Bavarian Red Cross. The blood donor sera were collected before the outbreak of the SARS-CoV-2 pandemic, i. e. before November 2019.

**B. Supplementary Methods**

**B 1. Precision and reproducibility of the immunoassay**

**B.1.1 Intra-lot (within-run) precision**. Intra-lot (within run) precision was determined with one lot of test strips, one dynablot processor, 2 operators with one experiment each on the same day. 4 IgG-positive specimen were tested 10 or 11 times each in one experiment in recomLine SARS-CoV-2, respectively. Variation coefficient of positive sera or sera above 25 grey intensities was below 19.9%

NP_HKU1 and NP_OC43 were also included in the data set and comply with the specification above (Vc below ≤20%).

**B.1.2. Intra-Lot (between-day) precision.** Intra-Lot (between-day) precision was determined for a panel of 4 samples (high positive, low positive, greyzone, negative). The serea were assayed in one lot as 4 fold replicates, per day, assayed on 3 different days, assayed by 2 operators, one lot of test kit, 2 dynablot processors.

The variation coefficient of positive sera or sera above 50 grey scales was <21.5%

**B.1.3.Interlot (between-day) precision**. Interlot (between-day) precision was determined with a panel of 4 samples (high positive, low positive, greyzone, negative) on 3 different days (3 lots of test kits, 2 dynablot processors) as 4 fold replicates per day, assayed by 2 operators. The variation coefficient of positive sera or sera above 50 grey intensities was <18% .

**B.2. Diagnostic sensitivity and specificity of the immunoassay**

**Sensitivity** of the recomLineSARS-CoV-2 assay was determined using the sera from 54 patients with PCR-confirmed SARS-CoV-2 infection. Sensitivity was 85.7 % for the time span < 12 days after onset of clinical symptoms, 95.2 % for the time span between 12 and 23 days and 100 % for sera taken later than 23 days after the onset of clinical symptoms.

**Specificity** of the recomLineSARS-CoV-2 assay was determined using a) 300 sera from healthy blood donors (taken before the pandemic), b) 191 sera with potential crossreactivity due to infection with seasonal coronaviruses, respiratory syncytial virus, influenza A and B, adenovirus, mycoplasma, chlamydia, EBV (IgM-positive), CMV (IgM-positive), anticellular reactivity (ANA/ENA) or pregnancy, c) 78 sera with possible interfering potential (lipemic, icteric, hemolytic sera and sera containing rheumatoid factor). The determined specificities were 99,7 % for a, 97,9 % for b and 97,5 % for c.

**C. Supplementary Results**

**C.1. Kinetics of avidity maturation of IgG towards S1 and RBD.**

Figure 1 A,B in the main manuscript demonstrates the kinetics of avidity maturation of IgG directed towards SARS-CoV-2 nucleoprotein (NP) and receptor-binding domain (RBD) for 15 sera taken from PCR-confirmed Covid-19 patients, using variable urea concentrations for avidity determination with the recomLine SARS-CoV-2 assay. Supplementary Figure 1 shows that the results obtained for avidity

Supplementary Figure 1


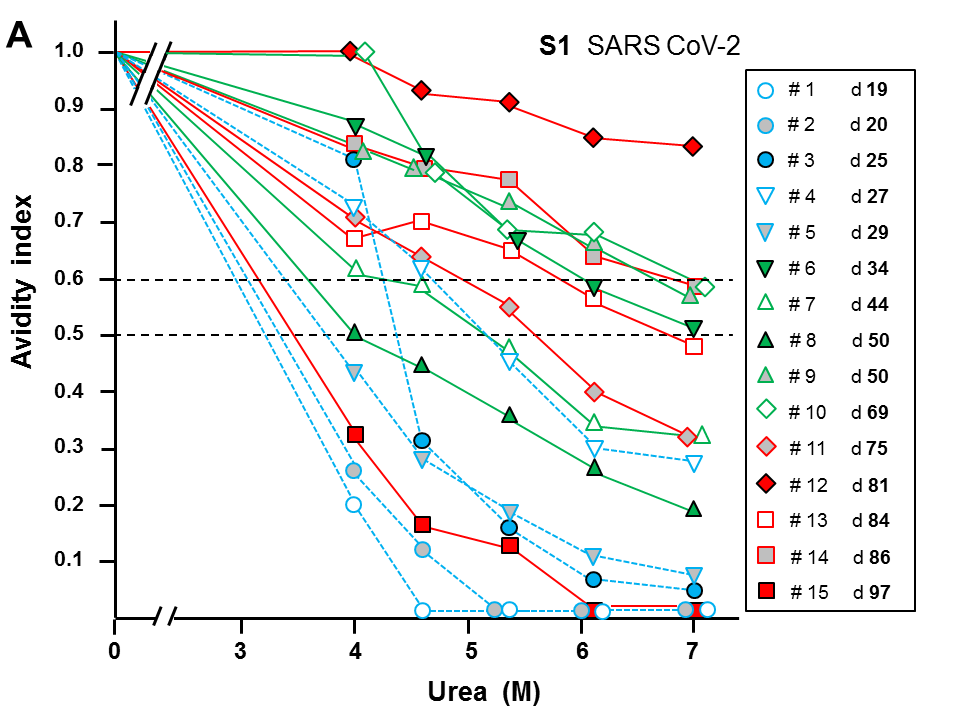

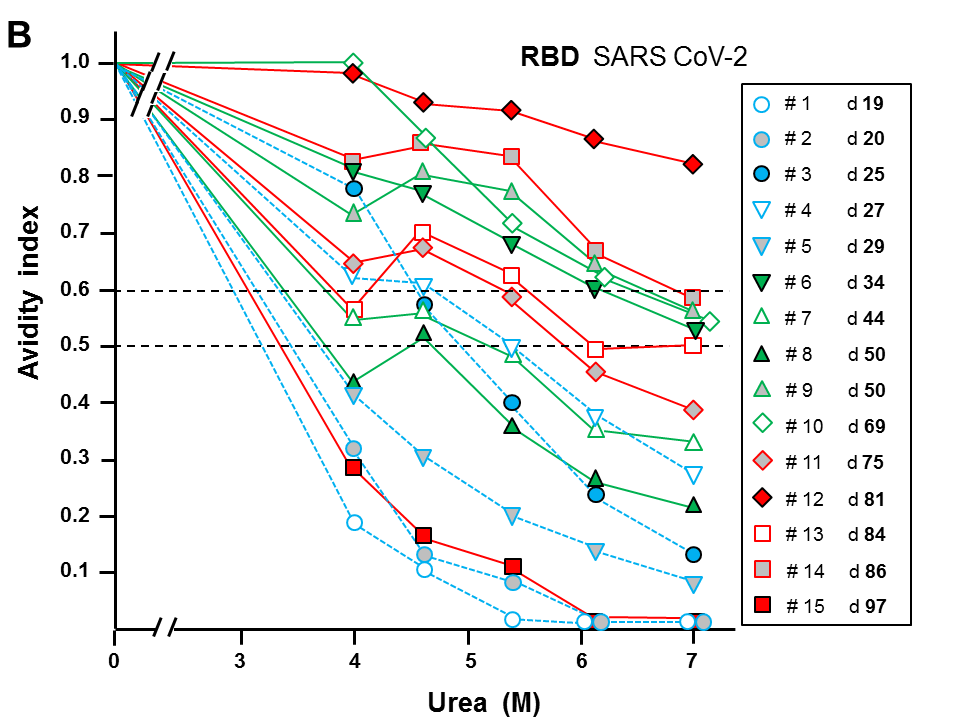


**Legend to Supplementary Figure 1:** **Avidity determination of IgG towards the spike protein S1 and receptor-binding domain (RBD) of SARS-CoV-2 in 15 sera from patients with COVID-19.**

Avidity determination was performed with the indicated concentrations of urea for IgG directed towards SARS-CoV-2 S1 (**A**) or RBD (**B**) in 15 sera of patients with COVID-19 and SARS-CoV-2 infection proven by PCR. The sera had been taken at varying times after the onset of disease, as indicated in the figure. Dashed lines indicate the level between low avidity (avidity index < 0.5), borderline avidity (avidity index between 0.5 and 0.6) and high avidity (avidity index >0.6). With the exception of one serum, the majority of sera exhibited IgG of low or borderline avidity for IgG towards S1 (A) and RBD (B), confirming the immature avidity response after SARS-CoV-2 infections. Though the avidity indices obtained at 7 M urea are mostly in the borderline and low avidity range, the titration with varying concentrations of urea visualizes the individual differences in a more pronounced and characteristic mode. This refined measurement therefore should allow to follow subtle changes of avidity in defined cases of analysis.

maturation of IgG towards SARS-CoV-2 S1 are nearly identical to those of IgG towards SARS-CoV-2 spike protein S1. These data confirm the frequent incomplete avidity maturation after SARS-CoV-2 infection.

Grey intensity units represent the respective IgG concentrations. When they were plotted against the days after onset of disease, no direct correlation was found (Supplementary Figure 2 A). In contrast, avidity indices obtained for IgG towards NP showed a direct correlation with the time after onset of disease, independent of the urea concentrations that had been used for analysis, i. e. 7 M under B and 5.3 M under C. Despite overall low avidity, due to incomplete avidity maturation, these data can be used to differentiate between early and late infection with SARS-CoV-2. Using an avidity index of 0.3, a discrimination between the first 50 days after onset of disease and later time points can be reached.

Finally, Supplementary Figure 3 illustrates that the avidity indices for IgG towards RBD have the tendency to be higher and more variable, compared to the avidity indices measured for IgG towards NP.

Supplementary Figure 2


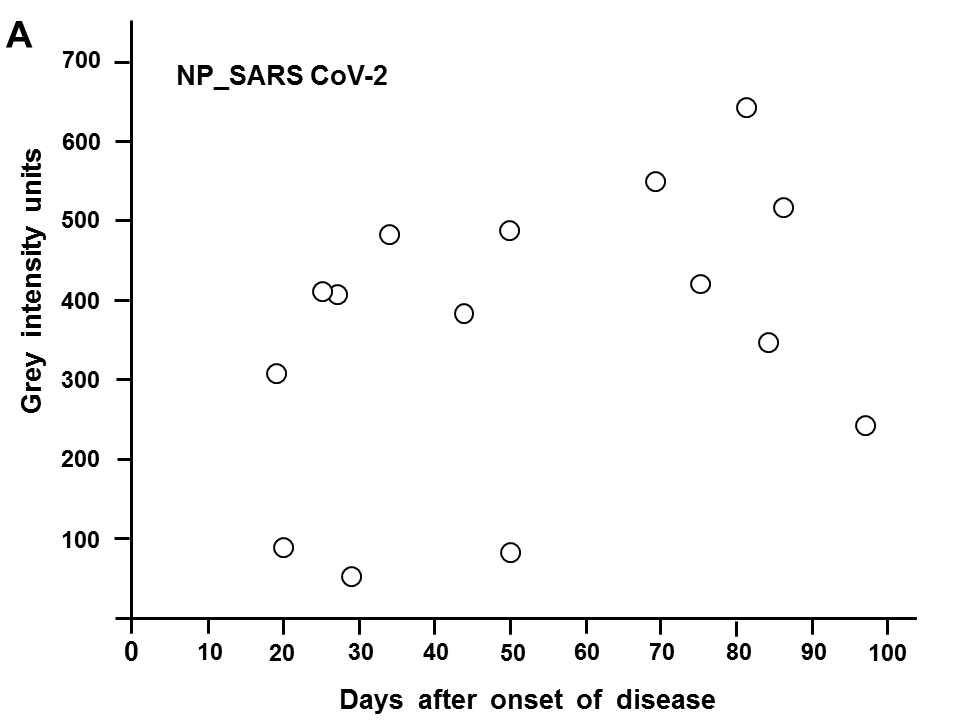

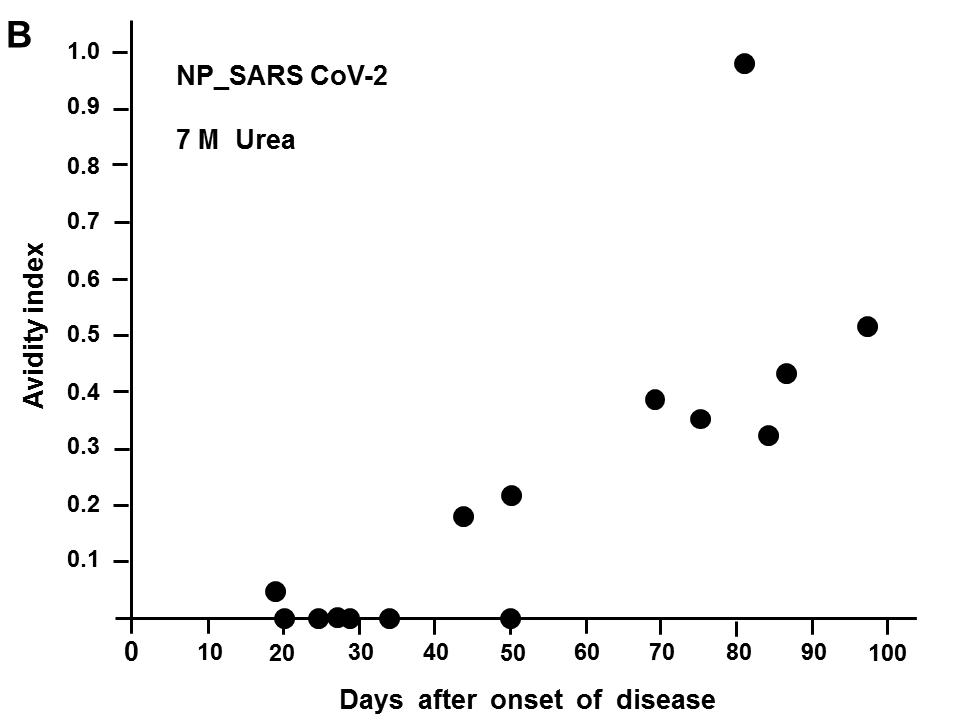

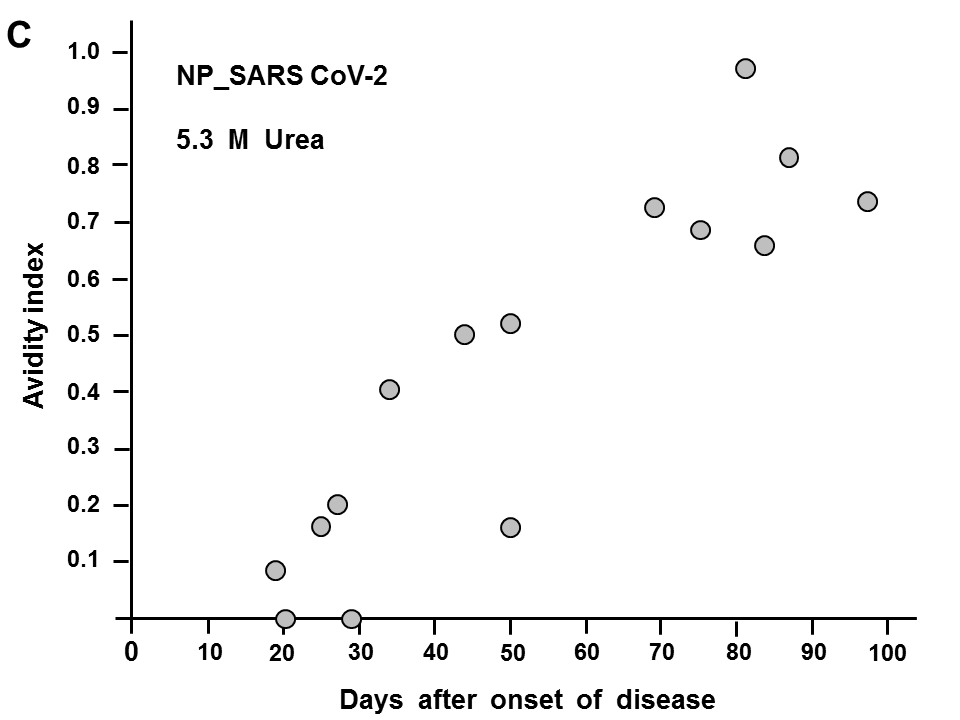


**Legend to Supplementary Figure 2: Grey intensity units and avidity indices with respect to the time after onset of disease**. The data have been taken from the experiment described in Figure 1 in the main text. Plotting of the time after onset of disease versus grey intensity units of IgG towards SARS-CoV-2 NP shows no correlation, whereas plotting of the corresponding avidity indices for IgG towards NP of SARS-CoV-2 versus time after onset of disease shows a direct correlation.

Supplementary Figure 3


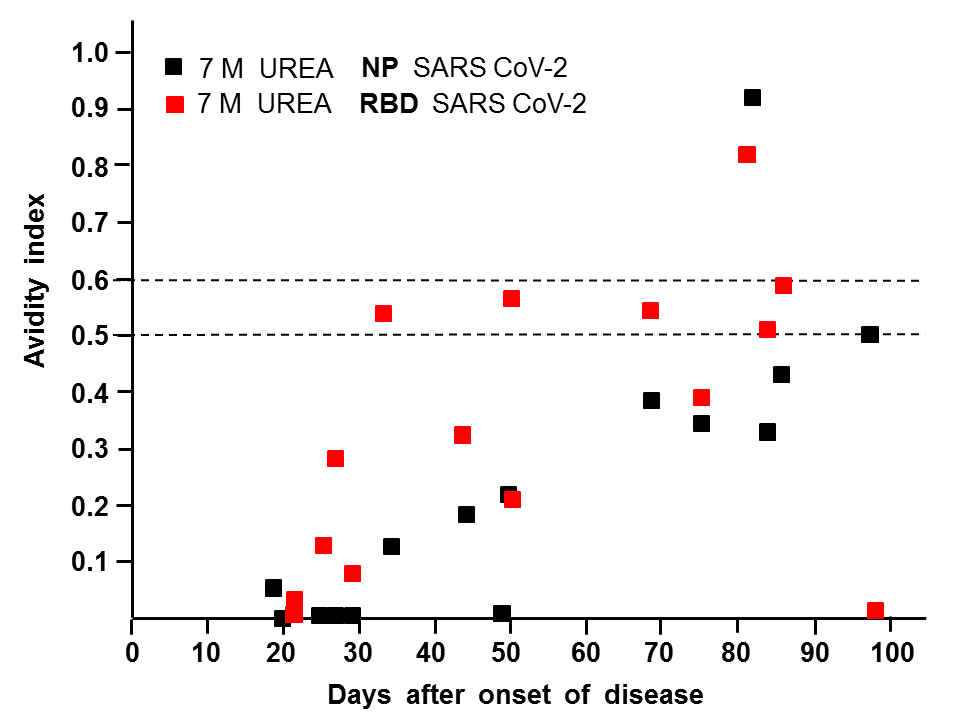


**Legend to Supplementary Figure 3: Comparison of the avidity indices for IgG directed towards SARS-CoV-2 NP and RBD with respect to the time after onset of disease.** The data have been taken from the experiment described in Figure 1 in the main manuscript. They show that the avidity indices for IgG towards SARS-CoV-2 RBD have the tendency to be higher and more variable than those determined for IgG towards SARS-CoV-2 NP. These differences do, however, not reach statistical significance.

**C.2 Verification of the key findings with larger number of sera from SARS-CoV-2-infected outpatients.**

The data presented so far in this manuscript and its supplement indicate that the humoral immune response towards SARS-CoV-2 infection seems to be characterized by frequent incomplete avidity maturation of IgG directed towards NP, RBD and S1. High avidity of IgG developed several months after onset of disease seemed to be the exception. This finding is completely opposite to the findings for other viruses, where avidity maturation is a regularly occurring process and lack of avidity maturation seems to be the exception.

Based on the more laborious method of titration with varying urea concentrations and the limitation of available sera, our initial approach was performed with a relatively low number of patients and sera. For evaluation of the significance of our findings and conclusions, the analysis of avidity maturation was extended to 90 sera. This analysis was performed under the conditions of routine diagnostics, i. e. by application of 7 M urea. The results obtained in this additional study were compared to the values obtained for treatment with 7 M urea as shown in the previous Figures 1-3, as well as Supplementary Figures 1-3.

Supplementary Figure 4 shows that the central findings of our study (using 15 sera) can be verified with the follow-up study, using 93 sera. The following statements are confirmed by both studies:

i) About 50-70 % of all sera showed an extremely low avidity index of less than 0.3 for the IgGs directed towards NP, RBD and S1.

ii) The percentage of sera with an avidity index between 0.3 and 0.5 was always lower than the percentage of sera with an avidity index <0.3.

iii) The percentage of sera with low avidity (avidity index up to 0.5) was always higher than the percentage of sera with avidity indices above 0.5.

iv) The percentage of sera with very high avidity (avidity index >0.6) was always less than 10 %, irrespective of the targeted antigen and the number of sera tested.

Therefore, the conclusion on incomplete avidity maturation after SARS-CoV-2 infection has been verified by the follow-up study with larger number of sera.

As avidity increases with time, the results shown in Supplementary Figure 4 were further analyzed with respect to time after onset of disease, in Supplementary Figure 5. Supplementary Figure 5 confirms our previous findings and conclusions and illustrates how avidity determination of IgG towards SARS-CoV-2 NP can be used for the differentiation between acute and past infection.

Supplementary Figure 4


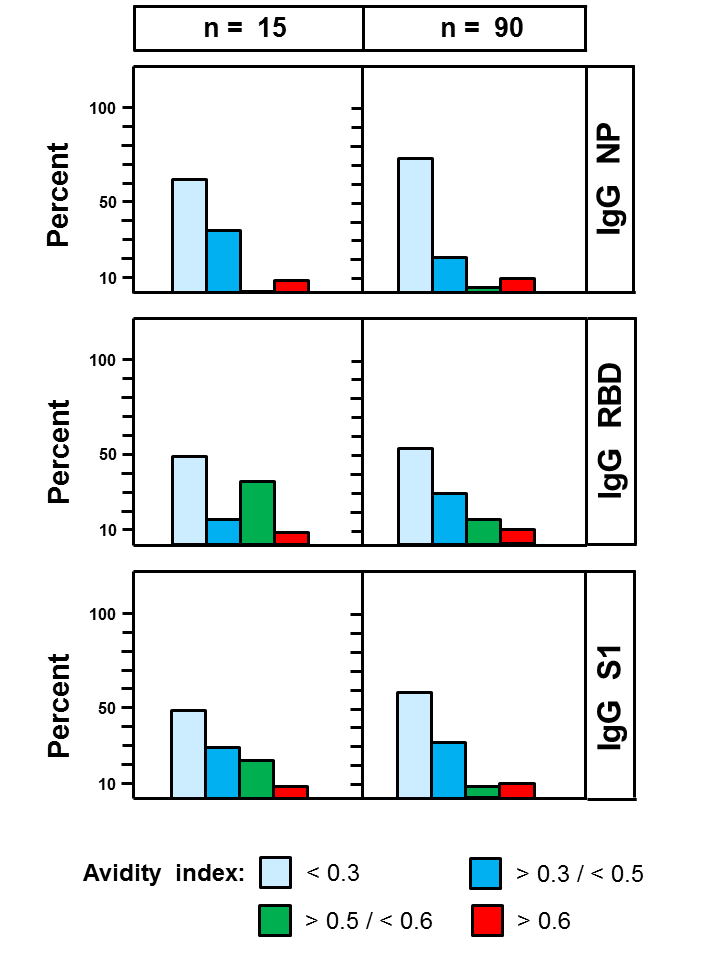


Legend to Supplementary Figure 4. **Verification of our study through increase in the number of tested sera**.

The avidity indices for IgG towards SARS-CoV-2 NP, RBD and S1 as obtained in our study, using 15 sera of adult Covid-19 outpatients and 7M urea (left side) were compared to the results obtained in a follow-up study with 93 sera from 70 adult Covid-19 outpatients (33 women, 37 men) tested under the same conditions (right side). The high prevelance of sera with low avidity, as well as the frequent incomplete avidity maturation were verified.

Supplementary Figure 5
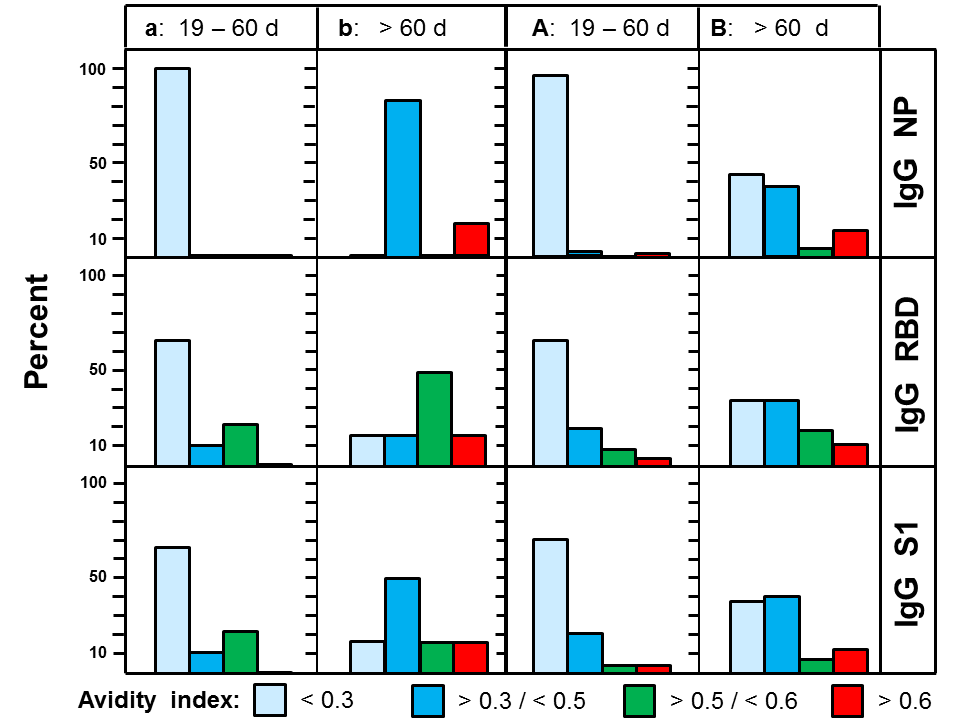


Legend to Supplementary Figure 5. **Time dependence of avidity maturation.**

The time dependence of avidity determination for 15 sera (a, b) and in a follow-up study with 93 sera (A, B) is demonstrated by differentiation of the avidity indices reached between 19 and 60 after onset of disease (a, A) versus 61-100 days (b, D) in the sera from Covid-10 outpatients with PCR-proven SARS-CoV-2 infection. The results confirm that despite incomplete avidity maturation, an increase in avidity with time is seen for the IgGs directed NP, RBD and S1. The avidity determination of IgG towards NP allows for a discrimination between early infection (less than 60 days after onset of disease) and past infection, when avidity indices of 0.3 are taken as discriminative line. If an avidity index of >0.3 is determined, an acute infection during the last 60 days is most unlikely. An avidity index of <0.3 indicates either acute infection or past infection with incomplete avidity maturation at a low level. Differentiation between these two possibilities can be obtained through the analysis of a follow-up serum to weeks later.

**C. 3. The potential impact of IgG towards seasonal coronaviruses on SARS-CoV-2 serology**

In addition to the examples shown in Figure 4 in the main manuscript, two more sera from PCR-confirmed Covid-19 patients were tested for IgG towards SARS-CoV-2 NP, RBD, S1 and IgG towards the NPs of four seasonal coronaviruses (229 E; NL63; OC43, HKU1), in the absence of presence of increasing concentrations of urea (Supplementary Figure 6). The analysis of the data obtained for the first patient (A-D) and the second patient (E-G) shows that the grey intensity units reached for IgG towards SARS-CoV-2 NP were always markedly higher than those reached for IgG towards the NPs of the seasonal coronaviruses. This finding further supports the data presented in Figure 4 in the main manuscript. It excludes that the signal for IgG towards SARS-CoV-2 NP is due to cross-reaction with IgG towards NP of one of the seasonal viruses. Both patients showed low avidity IgG towards NP of SARS-CoV-2 and high or low avidity IgG towards the NPs of seasonal coronaviruses. These findings are in line with our observation that incomplete avidity maturation of IgG is not restricted to the immune response towards SARS-CoV-2, but can also be observed for seasonal coronaviruses. Most likely, the low avidity indices of IgG towards NPs of seasonal viruses are not indicative of acute infection with these viruses in parallel to infection with SARS-CoV-2. They are rather indicative of an earlier infection with incomplete avidity maturation. The decisive distinction in individual cases can be obtained by kinetic analysis (Bauer et al., manuscript submitted). .

Supplementary Figure 6


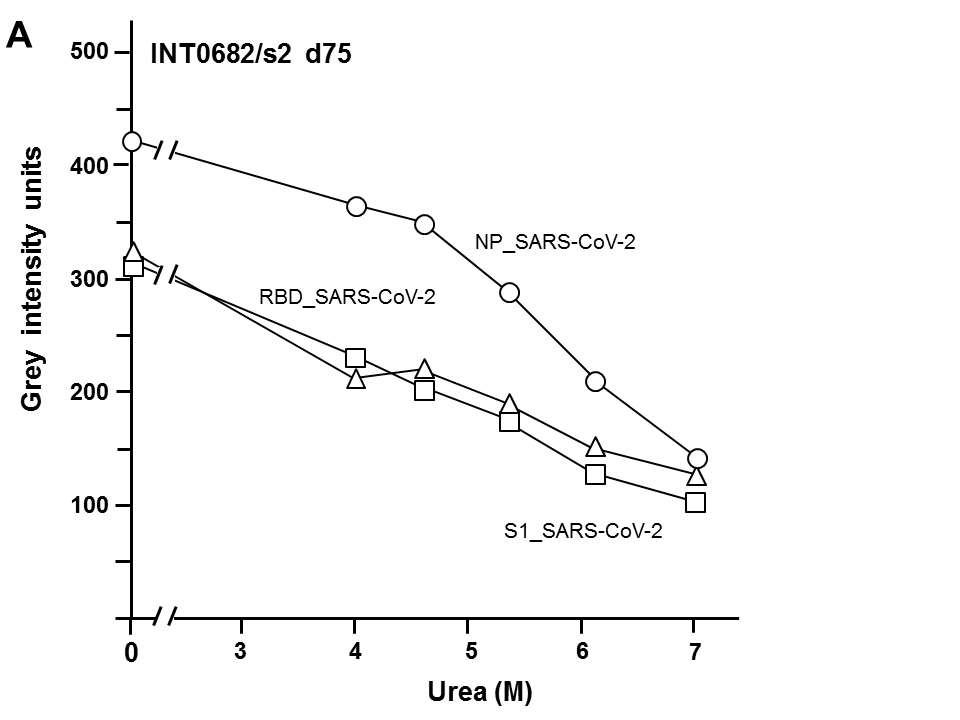

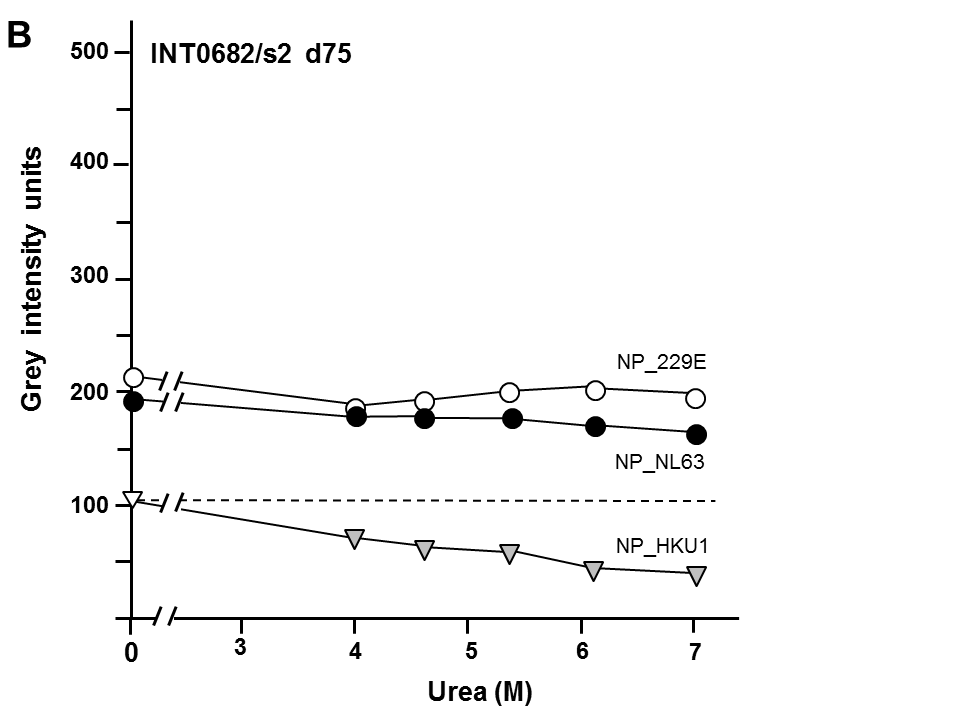

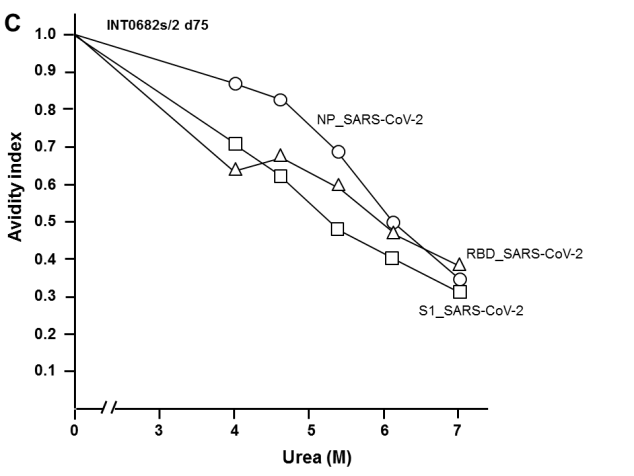

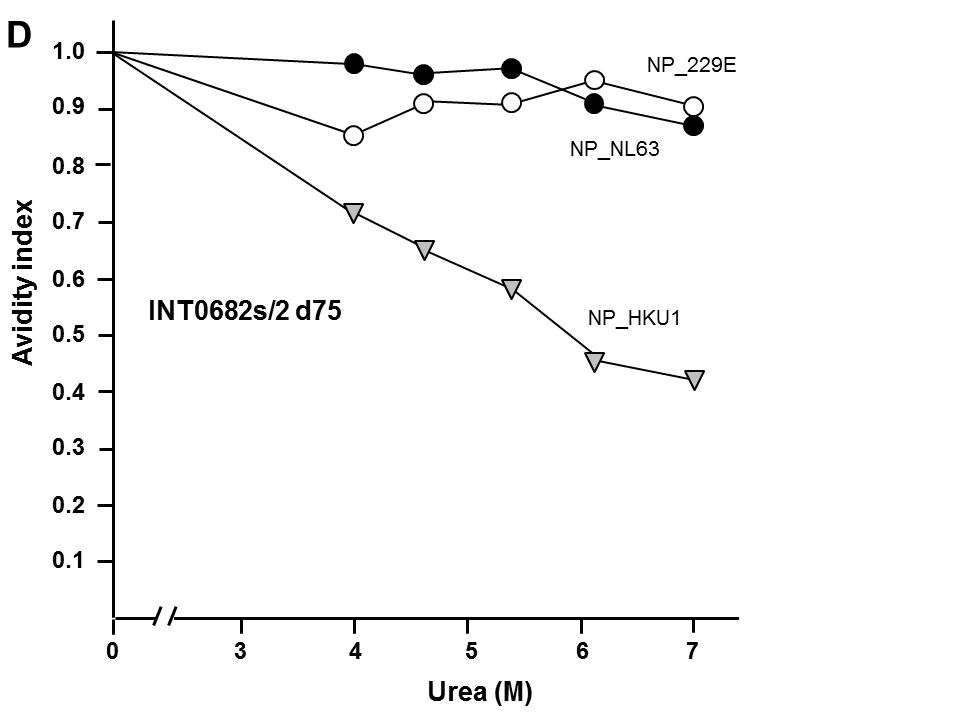

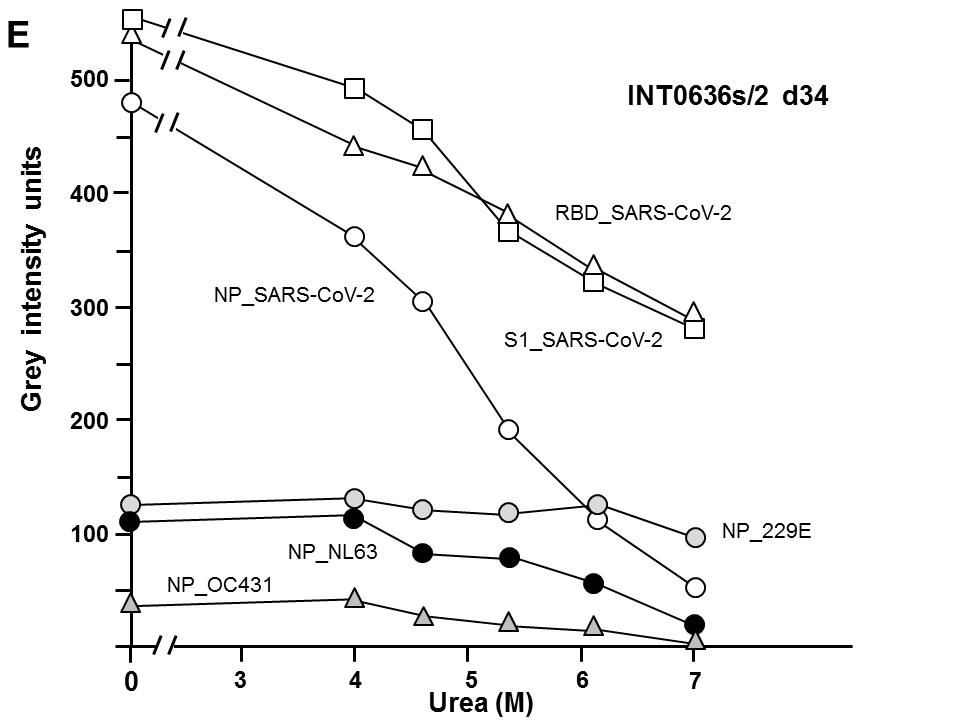

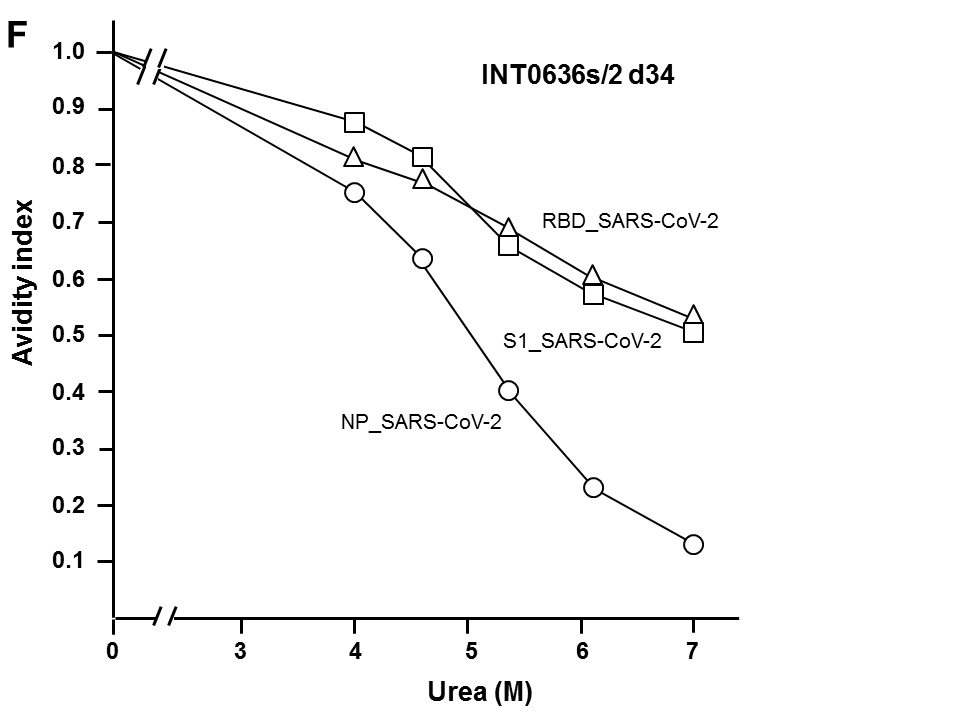

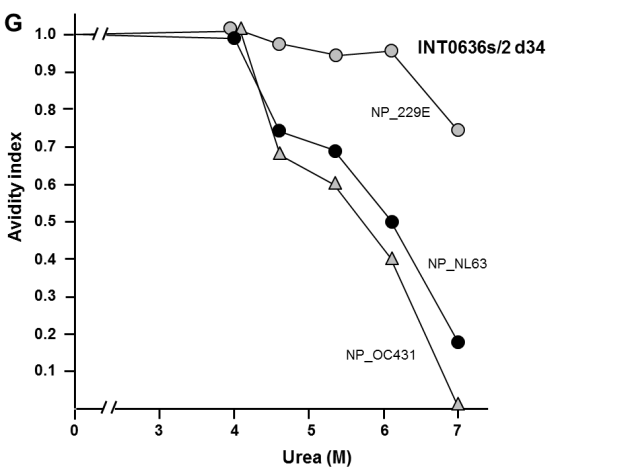


**Legend to Supplementary Figure 6: Comparison of grey intensity units and avidity indices obtained for IgG directed towards NP, RBD and S1 of SARS-CoV-2 and NP of four seasonal coronaviruses.** The sera from two patients with PCR-confirmed Covid-19 were tested for IgG towards NP, RBD, S1 of SARS-CoV-2 and IgG towards NP of four seasonal coronaviruses (229 E, NL63, OC43, HKU1), in the absence or presence of increasing concentrations of urea. The grey intensity units for patient #1 (A, B) and patient #2 (E), as well as the avidity indices for patient #1 (C, D) and patient #2 (F, G) are presented.

The figure shows that the grey intensity units obtained for IgG towards NP of seasonal coronaviruses are always markedly lower than those obtained for IgG towards NP of SARS-CoV-2 and therefore cannot explain the SARS-CoV-2 –specific signal as cross-reaction. Furthermore, the data in this figure confirm that incomplete avidity maturation can also be found for IgG towards the proteins of seasonal coronaviruses.

**D. Supplementary Discussion**

**D. 1. Potential causes and consequences of immature affinity (avidity)**

The establishment of immature avidity of IgG towards a virus, as shown for infections with SARS-CoV-2 and seasonal coronaviruses, is so far unique in virology. It raises many questions. Their resolution will also require rational and target-oriented serological testing.

Question number one is asking for the cause of immature avidity maturation after coronavirus infections, particularly those with SARS-CoV-2. There are two potential answers related to this aspect: 1) As affinity (avidity) maturation requires the sustained availability of the target antigen, which drives the selection process of B cells expressing IgG with higher affinity (avidity), a shortage of viral antigen accessible to the immune system might not allow sufficient cycles of selection to finally achieve high affinity (avidity) IgG. A similar scenario has been shown in other microbiological systems ^1^. Shortage of accessible antigen might be due to localized infection or down-modulation of viremia through induction of interferon responses. 2) The recognizable massive impact of SARS-CoV-2 infection on the immune system ^2^ might affect the process of affinity (avidity) maturation directly. This specific aspect awaits further clarification.

Question number two is asking for the biological consequences of immature affinity (avidity) of IgG directed towards SARS-CoV-2 and other coronaviruses. It seems reasonable to assume that immature affinity (avidity) might allow secondary infections and thus is one basis for repeated cycles of coronavirus infections in long-term intervals ^3, 4^. The scientific basis for this assumption are the numerous findings that show that the rare failures to reach high avidity IgG in other viral systems leads to an increased risk of reinfection and disease ^5-14,^ and that the efficiency of vaccination increases with the establishment of high avidity neutralizing IgG ^15, 16^ .

Question number three is focusing on the medical impact of low avidity IgG towards SARS-CoV-2 antigens. Is this unexpected phenomenon the reason for the possibility of reinfections with SARS-CoV-2, which have already been observed ^17-20^ ? If so, would it then be possible to achieve herd immunity after natural infections with the virus? Based on the above summarized arguments and the findings for seasonal coronaviruses, induction of herd immunity through natural infection rather seems unlikely to occur.

Question number four is related to vaccination towards SARS-CoV-2. Based on the information on other virus system ^5-16^, it is justified to speculate that high avidity of neutralizing antibodies might be necessary to assure protective immunity. But can we expect induction of a protective, high avidity response after vaccination in the light of the finding that natural infection with SARS-CoV-2 obviously does not reach this goal? Our optimistic judgement on this aspect is yes – as i) the vaccination mode might be adjusted in a way to present antigen for avidity maturation at optimal concentration and for a sufficiently long time and ii) a negative impact on the immune system, affecting avidity maturation in a negative way, is not as likely after supply with defined antigens through vaccination compared to infection with functional virus.

These questions and their answers point to the necessity of specific monitoring of i) the specificity and concentration of IgG induced through vaccination; ii) the neutralizing potential of the induced IgG, i. e. its capability to recognize those epitopes on viral surface protein that are required for interaction with target cells, and iii) as a new, but essential aspect in this discussion, the avidity of the neutralizing antibodies. We propose the evidence-based speculation that the avidity of the neutralizing antibodies might be decisive in the competition between binding of virus to cells (leading to infection) or tight binding to IgG, resulting in protective immunity.

The following chapter shows the serological test principles and outcomes that most likely will be relevant in this context.

**D. 2. Reflections on the use of avidity determination for the monitoring of vaccination towards SARS-CoV-2**

Supplementary Figure 7 describes the immunoblot-based assay system of the recomLineSARS-CoV-2 assay which has been used in our study. People without SARS-CoV-2 infection (a) can be expected to show IgG towards the NP of seasonal coronaviruses, both a high or low avidity. Due to the high specificity of the test system, usually there will be no signal indicating IgG directed towards SARS-CoV-2 NP, RBD or spike protein S1. After infection with SARS-CoV-2 (b, c), most infected people will develop IgG towards SARS-CoV-2 NP, RBD and S1 which, according to our findings, will probably remain at low or borderline avidity even after a long time after the onset of disease (or infection, in clinically inapparent cases).

Supplementary Figure 7


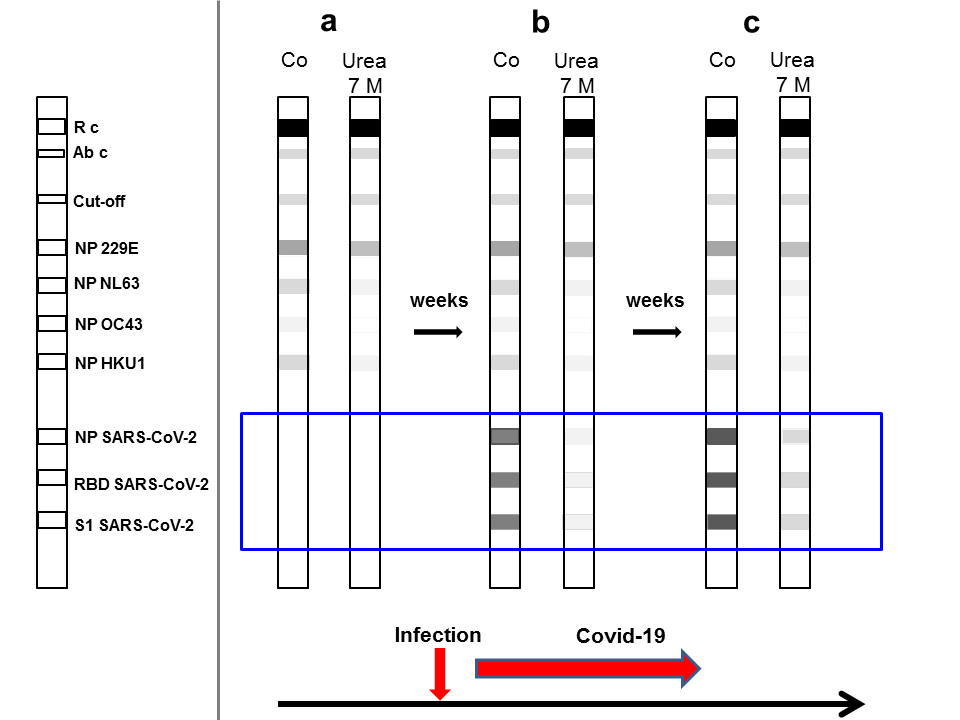


**Legend to Supplementary Figure 7. Avidity testing of IgG towards SARS-CoV-2 and seasonal coronaviruses.** The figure illustrates the test system that has been used in our study and that should be suitable to monitor the success of vaccination. On the left side, we see the arrangements of antigens and controls. Rc (reaction control) ensures that the test has been properly performed. Ab c (antibody control) ensures that IgG has been detected. The cut-off control is the basis for quantitative measurement. The test strip contains sufficient amount of highly purified recombinant NP of the seasonal coronaviruses 229 E, NL63; OC43, HKU1, and NP, RBD and S1 from SARS-CoV-2. Avidity is determined by the use of two parallel strips for each serum. After the first incubation step between serum and the test antigens, one strip is washed with buffer only, whereas the other is treated with 7 M urea for 3 min and is washed. The urea step removes low avidity IgG. See more details under Methods in the main article. Sera from persons not infected with SARS-CoV-2 can be expected not to induce a signal for IgG towards SARS-CoV-2 antigens, but to show IgG directed towards NP of seasonal coronaviruses, at high or low avidity (a). Low avidity may indicate acute infection with the respective virus or incomplete avidity maturation. After infection with SARS-CoV-2 and possible subsequent Covid-19 (b, c), most patients develop IgG towards all three SARS-CoV-2 antigens of low or intermediate avidity.

As low avidity IgG towards SARS-CoV-2 antigens is the typical response ^21-25^, the risk of reinfection might be high. Reinfection might be associated with the risk to develop Covid-19 again, or it may lead to a clinically inapparent state. It may be speculated that the second challenge with SARS-CoV-2 might drive avidity maturation of anti-SARS-CoV-2 IgG to a higher level of maturation and thus establish a state of protective immunity.

Immunization hopefully leads to the induction of IgG directed towards the relevant antigens RBD and S1, which mature to high avidity with time, as shown in Supplementary Figure 8.

Supplementary Figure 8


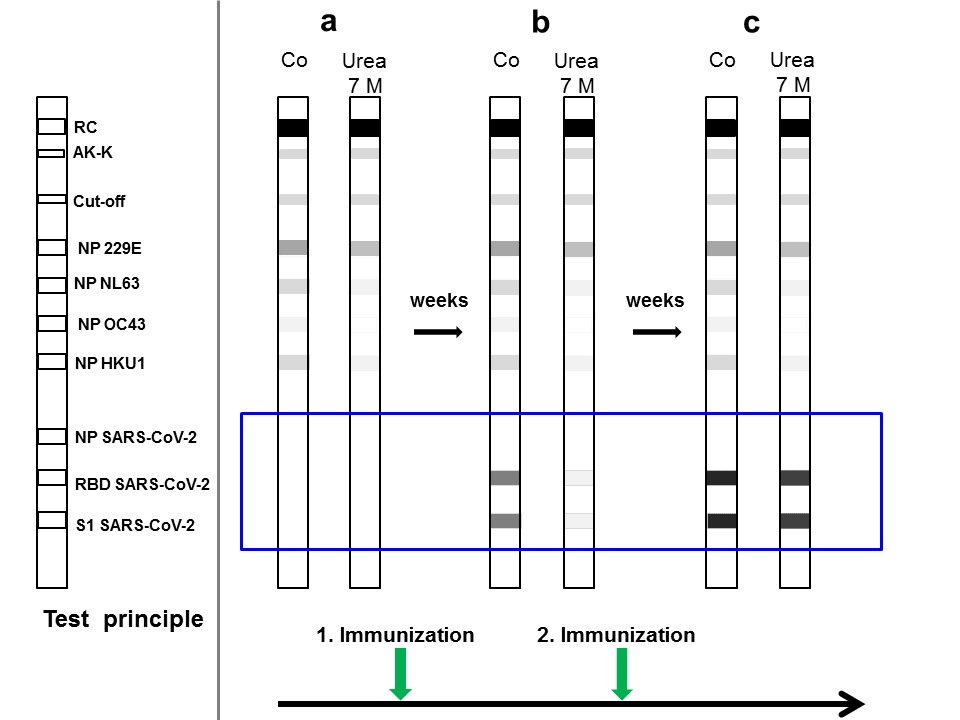


**Legend to Supplementary Figure 8. Optimal scenario for IgG responses after vaccination towards SARS-CoV-2.**

Persons not infected with SARS-CoV-2 do not show IgG towards SARS-CoV-2 antigens, but towards seasonal coronaviruses (a). After vaccination against SARS-CoV-2, low avidity IgG towards RBD and S1 is first induced (b). The second immunization step induces further maturation of avidity to high values (c), which is speculated to ensure protective immunity.

The test system presented in this paper is ready to verify or falsify this hypotheses.

After succesfull vaccination, characterized by a sufficiently high concentration of high avidity IgG directed towards SARS-CoV-2 RBD and S1, protective immunity seems to be likely.

A new contact with SARS-CoV-2 might then have no medical impact for the recipient. If sterile immunity has been established through vaccination, no immunological foot print of this novel viral encounter is expected, as illustrated in Supplementary Figure 9. However, if vaccination did not result in sterile immunity, a novel contact between SARS-CoV-2 and the vaccinated person, harbouring high avidity IgG towards SARS-CoV-2, might allow restricted replication of the virus. Thiswould become detectable through the induction of low avidity IgG towards NP, but the establishment of disease would be minimized (Supplementary Figure 10). An important remaining question, here, is whether a person infected according to this scenario would be able to transmit virus for a limited time.

Supplementary Figure 9


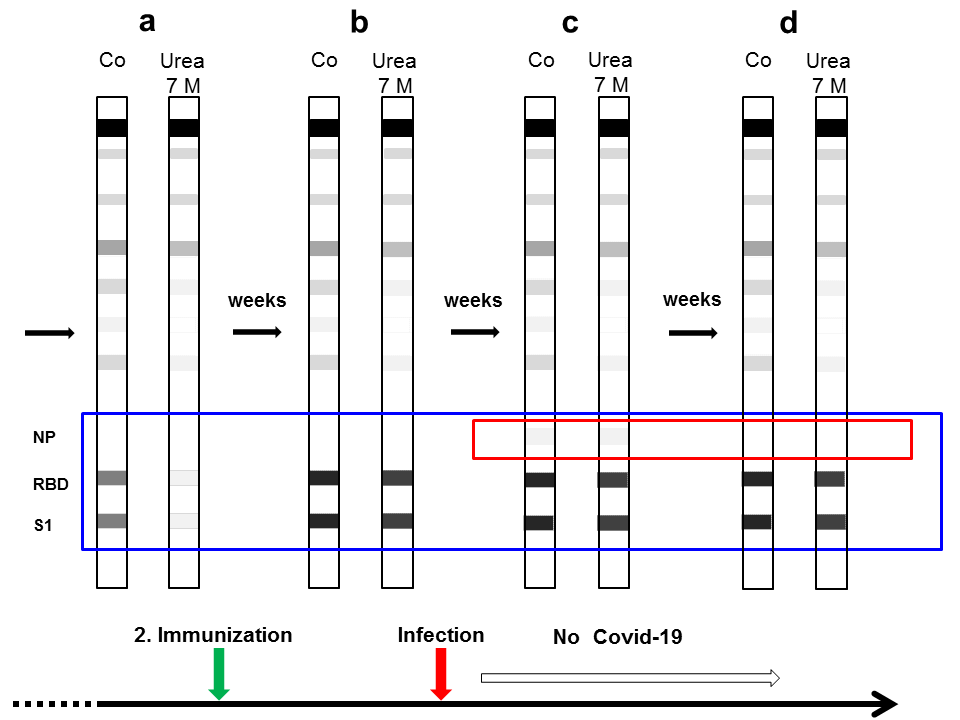


**Legend to Figure 9. Prevention of Covid-19 through protective immunity.**

If high avidity of IgG towards RBD and S1 has been achieved through immunization (a, b), a contact with infectious SARS-CoV-2 will possibly not result in Covid-19. If sterile immunity had been achieved through vaccination, even no immunological footprint of the potentially infecting virus (appearance of IgG towards NP) will become detectable (c, d).

Supplementary Figure 10


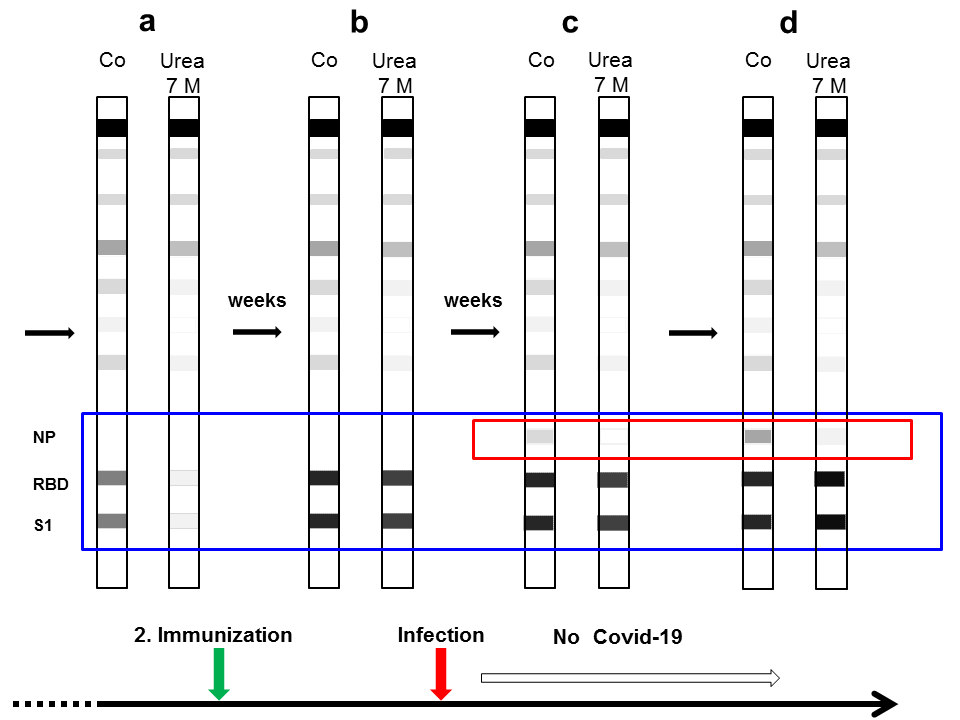


**Legend to Supplementary Figure 10. Alternative scenario: immunization protects towards disease, but allows for limited virus replication.** If immunization does not lead to sterile immunity despite high avidity of IgG towards RBD and S1 (a, b), Covid-19 will be most likely prevented after infection with SARS-CoV-2, but limited viral replication might be possible. This may result in the induction of low avidity IgG towards NP (c, d).

If however, no high avidity IgG towards RBD and S1 was established despite two immunization steps, and therefore no protective immunity can be expected, infection with SARS-CoV-2 with the risk of Covid-19 are possible. The combination of incomplete avidity maturation after vaccination and subsequent SARS-CoV-2 infection has a chance to drive avidity maturation of IgG towards RBD and S1 to avidity higher level.

Vaccination of large numbers of people in the present pandemic situation will most likely also lead to vaccination of people who had encountered clinically inapparent SARS-CoV-2 infection before the vaccination. The serological consequences for this specific situation can be predicted as follows: It can be expected that in most cases, the clinically inapparent SARS-CoV-2 infection will cause induction of low avidity IgG towards SARS-CoV-2 NP, RBD and S1. Subsequent vaccination most likely enhances IgG production as well as avidity maturation of IgG directed towards RBD and S1, whereas no effect on IgG towards NP can be expected. As a result, protective immunity can be speculated to be established and therefore reinfection would not cause Covid-19 or even would be completely prevented, if sterile immunity was achieved. Alternatively, the low avidity IgG established through primary contact with SARS-CoV-2 might interfere with the vaccination process by removing antigen provided through the vaccination process. In this case, the proband would remain in an unprotected state and encounter the risk of clinically apparent SARS-CoV-2 infection. The precise measurement of IgG responses and their avidity has the potential to discriminate between these theoretical scenarios in individual cases.

Based on these findings and considerations, we suggest to use avidity testing of IgG towards SARS-CoV-2 NP, RBD, S1 and parallel determination of NP towards seasonal coronaviruses to monitor the success of vaccine application, in order to optimize the vaccination scheme ^26^. Furthermore, individual testing of IgG avidity related to the response towards RBD and S1 might be useful to determine the likelihood of protective immunity induced by vaccination. These individual avidity determinations may be especially relevant for people at high risk and for people with professions that imply frequent contacts with persons that are potentially transmitting SARS-CoV-2.

**E. Supplementary References**

1 Goldblatt D, Pinto Vaz A R J P M, Miller E. Antibody Avidity as a surrogate marker of successful priming by *Haemophilus influenzae* type b conjugate vaccines following infant immunization. *J Inf Dis* 1998; 177, 1112–1115.

2 [Zhou R,](https://www.sciencedirect.com/science/article/pii/S1074761320303332#!) [To KK-W,](https://www.sciencedirect.com/science/article/pii/S1074761320303332#!) [Wong Y-C,](https://www.sciencedirect.com/science/article/pii/S1074761320303332#!) [Liu L,](https://www.sciencedirect.com/science/article/pii/S1074761320303332#!) [Zhou B,](https://www.sciencedirect.com/science/article/pii/S1074761320303332#!) [Li X,](https://www.sciencedirect.com/science/article/pii/S1074761320303332#!) [Huang H,](https://www.sciencedirect.com/science/article/pii/S1074761320303332#!) [i Mo Y,](https://www.sciencedirect.com/science/article/pii/S1074761320303332#!) [Luk T-Y,](https://www.sciencedirect.com/science/article/pii/S1074761320303332#!) [Lau TT-K,](https://www.sciencedirect.com/science/article/pii/S1074761320303332#!) [Yeung P,](https://www.sciencedirect.com/science/article/pii/S1074761320303332#!)  [Chan W-M,](https://www.sciencedirect.com/science/article/pii/S1074761320303332#!) [Wu AK-L,](https://www.sciencedirect.com/science/article/pii/S1074761320303332#!) [Lung K-C,](https://www.sciencedirect.com/science/article/pii/S1074761320303332#!) [Tsang OT-Y,](https://www.sciencedirect.com/science/article/pii/S1074761320303332#!) [Leung W-S,](https://www.sciencedirect.com/science/article/pii/S1074761320303332#!) [Hung IF-N,](https://www.sciencedirect.com/science/article/pii/S1074761320303332#!) [Yuen K-Y,](https://www.sciencedirect.com/science/article/pii/S1074761320303332#!)[.](https://www.sciencedirect.com/science/article/pii/S1074761320303332#!) Acute SARS-CoV-2 Infection Impairs Dendritic Cell and T Cell Responses. *Immunity* 2020; 53: 864-877**.**

3 Edridge AWD, Kaczorowska J, Hoste ACR, Bakker M, Klein M, Loens K, Jebbink MF, Matser A, Kinsella CM, Rueda P, Ieven M, Goossens H, Prins M, Sastre P, Deijs M, van der Hoek L. Seasonal corona virus protective immunity is short-lasting. Nature Medicine 2020; 26: 1691-1693.

4 Galanti M and Shaman J. Direct observation of repeated infections with endemic coronaviruses*.* J Infect Dis 2020; jiaa392. <https://doi.org/10.1093/infdis/jiaa392>

5 Junker AK, Tilley P. Varicella-zoster virus antibody avidity and subclass patterns in children with recurrent chickenpox. *J Med Virol* 1994; 43: 119-124.

6 Martin KA, Junker AK, Thomas EE, Van Allen MI, Friedman, JM. Occcurence of chickenpox during pregnancy in women seropositive for varicella-zoster virus. *J Infect Dis* 1994; 170: 991-995.

7 Boppana SB, Britt WJ. Antiviral antibody responses and intrauterine transmission after primary maternal cytomegalovirus infection. *J Infect Dis* 1995; 171: 1115-1121.

8 Lazzarotto T, Varani S, Spezzacatena P, Gabriell L, Pradelli P, Guerra B, Landini MP. Maternal IgG Avidity and IgM Detected by Blot as Diagnostic Tools to Identify Pregnant Women at Risk of Transmitting Cytomegalovirus. *Virol Immunol* 2009; 13: 137-141. <https://doi.org/10.1089/vim.2000.13.137>

9 Seo S, Cho Y, Park J. [Serologic screening of pregnant Korean women for primary human cytomegalovirus infection using IgG avidity test.](https://pubmed.ncbi.nlm.nih.gov/20046088/) *Korean J Lab Med*. 2009; 29:557-62. doi: 10.3343/kjlm.2009.29.6.557. PMID: 20046088

10 Kaneko M, Ohhashi M, Minematsu T, Muraoka J, Kusumoto K, Sameshima H. [Maternal immunoglobulin G avidity as a diagnostic tool to identify pregnant women at risk of congenital cytomegalovirus infection.](https://pubmed.ncbi.nlm.nih.gov/28034524/) *J Infect Chemother* 2017; 23:173-176. doi: 10.1016/j.jiac.2016.12.001. Epub 2016 Dec 26. PMID: 28034524

11 Kontio, M., Jokinen, S., Paunio, M., Peltola, H.& Davidkin, I. Waning antibody levels and avidity: implications for MMR vaccine-induced protection. *J Inf Dis* 2012; 206: 1542-1548.

12 Paunio M, Hedman K, Davidkin I, Peltola H. IgG avidity to distinguish secondary from primary measles vaccination failures: prospects for a more effective global measles elimination strategy. *Exp Opininion Pharmacotherapy* 2005; 4: 1215-1225.

13 Puschnik A, Lau L., Cromwell EA., Balmaseda A, Zompi S, Harris E. Correlation between Dengue-specific neutralizing antibodies and serum avidity in primary and secondary Dengue virus 3 natural infections in humans.*PLOS Neglected Tropical Diseases* 2013; 7: e2274

14 Delgado MF, Coviello S, Monsalvo AC, Melendi GA, Hernandez JZ, Batalle JP, Diaz L, Trento A, Chang H-Y, Mitzner W, Ravetch J, Melero JA, Irusta PM, Polack FP. Lack of antibody affinity maturation due to poor Toll-like receptor stimulation leads to enhanced respiratory syncytial virus disease. *Nature Medicine* 2009; 15: 34-41.

DOI 10.1099/jgv.0.001439

15 Lai L, Vödrös D, Kozlowski PA, Montefiori DC, Wilson RL, Akerstrom VL, Chennareddi L, Yu T, Kannanganat S, Ofielu L, Villinger F, Wyatt LS, Moss B, Amara RR, Robinson HL. GM-CSF DNA: An adjuvant for higher avidity IgG, rectal IgA, and increased protection against the acute phase of a SHIV-89.6P challenge by a DNA/MVA immunodeficiency virus vaccine. *Virology* 2007; 369: 153–167

16 Pegu P, Vaccari M, Gordon S, Keele BF, Doster M, Guan Y, Ferrari G, Pal R, Ferrari MG, Whitney S, Hudacik L, Billings E, Rao M, Montefiori D, Tomaras G, Alam SM, Fenizia C, Lifson JD, Stablein D, Tartaglia J, Michael N, Kim J, Venzon D, Franchinia G. Antibodies with high avidity to the gp120 envelope protein in protection from Simian Immunodeficiency Virus SIVmac251. Acquisition in an immunization regimen that mimics the RV-144 Thai Trial. *J Virol* 2013; 87: 1708–1719.

17 Overbaugh J. Understanding protection from SARS-CoV-2 by studying reinfection. *Nature Medicine* 2020; 26: 1678-1685.

# 18 [To](javascript:;) KK-W, [Hung](javascript:;) IF-N, [Ip](javascript:;) JD, [Chu](javascript:;) AW-C, [Chan](javascript:;) W-M, [Tam](javascript:;) AR, [Fong](javascript:;) CH-Y, [Yuan](javascript:;) S, [Tsoi](javascript:;) H-W, [Ng](javascript:;) AC-K, [Lee](javascript:;) LL-Y, [Wan](javascript:;) P, [Tso](javascript:;) EY-K, [To](javascript:;) W-K, [Tsang](javascript:;) DN-C, [Chan](javascript:;) K-HC, [Huang](javascript:;) J-D, [Kok](javascript:;) K-H, [Cheng](javascript:;) VC-C, [Yuen](javascript:;) K-Y. Coronavirus Disease 2019 (COVID-19) Re-infection by a Phylogenetically Distinct Severe Acute Respiratory Syndrome Coronavirus 2 Strain Confirmed by Whole Genome Sequencing. Clin Infect Dis 2020, ciaa1275, <https://doi.org/10.1093/cid/ciaa1275>.

19 Tillett RL, Sevinsky JR, Hartley PD, Kerwin H, Crawford N, Gorzalski A, Laverdure C, Verma SC, Rossetto CC, Jackson D, Farrell MJ, Van Hooser S, Pandori M. Genomic evidence for reinfection with SARS-CoV-2: a case study. *The Lancet* 2020. <https://doi.org/10.1016/S1473-3099(20)30764-7>

# 20 [Gupta](javascript:;) V, [Bhoyar](javascript:;) RC, [Jain](javascript:;) A, [Srivastava](javascript:;) S, [Upadhayay](javascript:;) R, [Imran](javascript:;) M, [Jolly](javascript:;) B, [Divakar](javascript:;) MK, [Sharma](javascript:;) D, [Sehgal](javascript:;) P, [Ranjan](javascript:;) G, [Gupta](javascript:;) R, [Scaria](javascript:;) V, [Sivasubbu](javascript:;) S. Asymptomatic Reinfection in 2 Healthcare Workers From India With Genetically Distinct Severe Acute Respiratory Syndrome Coronavirus 2. Clin Infect Dis 2020, ciaa1451, <https://doi.org/10.1093/cid/ciaa1451>

21 Strömer A, Grobe O, Rose R, Fickenscher H, Lorentz T, Krumbholz A. Diagnostic accuracy of six commercial SARS-CoV-2 IgG/total antibody assays and identification of SARS-CoV-2 neutralizing antibodies in convalescent sera. *medRxiv* 2020; doi: <https://doi.org/10.1101/2020.06.15.20131672>

22 Strömer A, Rose R, Grobe O, Neumann F, Fickenscher H, Lorentz T, Krumbholz A. Kinetics of nucleo- and spike protein-specific immunoglobulin G and of virus-neutralizing antibodies after SARS-CoV-2 infection. *Microorganisms* 2020, 8, 1572;

doi: 10.3390/microorganisms8101572

23 Liu T, Hsiung J, Zhao S, Kost J, Sreedhar D, Hanson CV, Olson K, Keare D, ChangST, Bliden KP, Gurbel PA, Tantry US, Roche J, Press C, Boggs J, Rodriguez-Soto JP, Montoya JG, Tang M, Dai H. Quantification of antibody avidities and accurate detection of SARS-CoV-2 antibodies in serum and saliva on plasmonic substrates. Nature Biomedical Engineering 2020; 4:1188-1196.

24 Benner S, Patel EU, Laeyendecker O, Pekosz A, Littlefield K, Eby Y, Fernandez RE, Miller J, Kirby CS, Keruly M, Klock E, Baker OR, Schmidt HA, Shrestha R, Burgess I, Bonny TS, Clarke W, Caturegli P, Sullivan D, Shoham S, Quinn TC, Bloch EM, Casadevall A, Tobian AAR, Redd AD. SARS-CoV-2 antibody avidity responses in covid-19 patients and convalescent plasma donors*.* J Inf Dis*,* 2020; jiaa581, <https://doi.org/10.1093/infdis/jiaa581>

25 Klein SL, Pekosz A, Park H-S, Ursin RL, Shapiro JR, Benner SE, Littlefield K, Kumar S, Naik HM, Betenbaugh MJ, Shrestha R, Wu AJ, Hughes RM, Burgess I, Caturegli P, Laeyendecker O, Quinn TC, Sullivan D, Shoham S, Redd AD, Bloch EM, Casadevall A, Tobian AAR.Sex, age, and hospitalization drive antibody responses in 1 a COVID-19 convalescent plasma donor population. *J Clin Invest*. 2020. <https://doi.org/10.1172/JCI142004>

26 Bauer G. The potential significance of high avidity IgG for protective immunity towards SARS CoV-2. Int J Infect Dis, in press.
